# A *CsTu*‐*TS1* regulatory module promotes fruit tubercule formation in cucumber

**DOI:** 10.1111/pbi.12977

**Published:** 2018-07-22

**Authors:** Sen Yang, Changlong Wen, Bin Liu, Yanling Cai, Shudan Xue, Ezra S. Bartholomew, Mingming Dong, Chen Jian, Shuo Xu, Ting Wang, Wenzhu Qi, Jinan Pang, Dehua Ma, Xingwang Liu, Huazhong Ren

**Affiliations:** ^1^ Beijing Key Laboratory of Growth and Developmental Regulation for Protected Vegetable Crops College of Horticulture China Agricultural University Beijing China; ^2^ Beijing Vegetable Research Center (BVRC) Beijing Academy of Agricultural and Forestry Sciences National Engineering Research Center for Vegetables Beijing China; ^3^ Tianjin Derit Seeds Co. Ltd Tianjin China

**Keywords:** cucumber, fruit tubercule size, auxin, *CsTu‐TS1* regulatory module

## Abstract

The fruit epidermal features such as the size of tubercules are important fruit quality traits for cucumber production. But the mechanisms underlying tubercule formation remain elusive. Here, tubercule size locus *CsTS1* was identified by map‐based cloning and was found to encode an oleosin protein. Allelic variation was identified in the promoter region of *CsTS1*, resulting in low expression of *CsTS1* in all 22 different small‐warty or nonwarty cucumber lines. High *CsTS1* expression levels were closely correlated with increased fruit tubercule size among 44 different cucumber lines. Transgenic complementation and RNAi‐mediated gene silencing of *CsTS1* in transgenic cucumber plants demonstrated that *CsTS1* positively regulates the development of tubercules. *CsTS1* is highly expressed in the peel at fruit tubercule forming and enlargement stage. Auxin content and expression of three auxin signalling pathway genes were altered in the *35S:CsTS1* and *CsTS1*‐RNAi fruit tubercules, a result that was supported by comparing the cell size of the control and transgenic fruit tubercules. CsTu, a C_2_H_2_ zinc finger domain transcription factor that regulates tubercule initiation, binds directly to the *CsTS1* promoter and promotes its expression. Taken together, our results reveal a novel mechanism in which the *CsTu‐TS1* complex promotes fruit tubercule formation in cucumber.

## Introduction

Cucumber (*Cucumis sativus*; 2n = 2x = 14) is an economically important vegetable crop cultivated around the world (Huang *et al*., 2009; Li *et al*., 2013; Yang *et al*., 2014). The cucumber fruit is a pepo that is covered with tubercules, trichomes and a thick cuticle (Roth, 1977; Wang *et al.,* 2015a). Usually, the larger trichomes on cucumber fruits are called spines and are composed of a stalk and a base (Chen *et al*., 2014). Tubercules are derived from several layers of cells that lie near the spine base; these cells divide and increase in number (Yang *et al*., 2014). In cucumber fruits, when tubercules are combined with spines, the result is a characteristic warty (Wty) trait, and on this basis, cucumber lines are divided into those with Wty and non‐Wty (nWty) fruits (Yang *et al*., 2014). The Wty trait provides an excellent model system to study plant differentiation. More importantly, the Wty trait is important for assessing cucumber appearance and commodity quality and it directly affects the market value (Yang *et al*., 2014). During the development of large‐warty (L‐Wty) cucumber fruit, tubercules and trichomes are randomly scattered on the fruit surface relative to the deep ridges along the length of the fruit (Ando *et al*., 2012; Chen *et al*., 2014). Compared with L‐Wty cucumber fruits, small‐warty (S‐Wty) or nWty fruits possess a smoother appearance and are more easily cleaned, packed, stored and transported. Moreover, S‐Wty and nWty fruits also have a more pleasant taste and lower quantities of pesticide residues (Li *et al*., 2015; Pan *et al*., 2015; Yang *et al*., 2014; Zhang *et al*., 2010). As a result, S‐Wty and nWty cucumber fruits are becoming increasingly popular worldwide, even in countries that have traditionally enjoyed L‐Wty cucumbers, and are gradually becoming desirable in cucumber breeding. Therefore, there is considerable interest in gaining a greater understanding of the molecular mechanisms underlying cucumber fruit tubercule development to enhance the economic value of cucumber production and breeding programmes.

Because there is no Wty fruit trait in the model plant *Arabidopsis thaliana*, the current research into the regulatory mechanism of fruit tubercule development is mainly focused on cucumbers. Previous genetic analyses have indicated that the cucumber nWty fruit trait is controlled by a single recessive nuclear allele, *cstu*, and that the cucumber *glabrous‐1* (*csgl1*) gene is epistatic to the *CsTu* gene (Cao *et al*., 2001; Yang *et al*., 2014). *CsTu* encodes a C_2_H_2_ zinc finger transcription factor (TF) and probably promotes cytokinin biosynthesis in fruit warts, thereby stimulating cell division and leading to the formation of fruit tubercules (Yang *et al*.,^2014^). We recently showed that the WD‐repeat protein CsTTG1 also regulates the development of the cucumber warty trait (Chen *et al*., 2016). Overexpression of *CsTTG1* increased the number of fruit tubercules, and the expression levels of *CsTu* increased in *35S:CsTTG1*‐overexpressing lines than in the wild type. It therefore meant that *CsTTG1* acts upstream of *CsTu* to regulate the tubercule formation (Chen *et al*., 2016). However, the regulatory network underlying the development of cucumber fruit tubercules remains poorly understood.

In this study, we report the identification and functional characterization of *CsTS1*, a key element controlling fruit tubercules formation in cucumber. Our research indicates that *CsTS1* positively regulates the development of tubercules and directly interact with CsTu, a C_2_H_2_ zinc finger domain transcription factor that regulates tubercule initiation.

In addition, our research also suggests that *CsTS1* controls the development of cucumber fruit tubercules through increasing auxin content. Taken together, these results presented here provide important insights into the role of *CsTu‐TS1* in the regulatory module controlling fruit tubercule formation in cucumbers.

## Results

### CsTS1 controls the size of cucumber fruit tubercules

**Figure 1:**
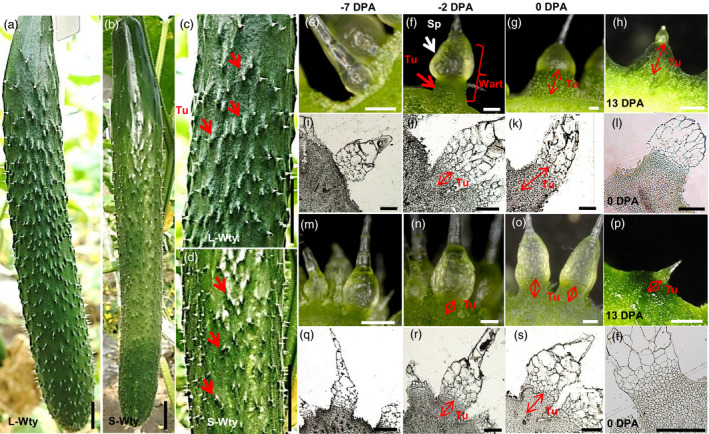
Morphological and cytological characterization of the L‐Wty fruit line 3546‐1 and the S‐Wty fruit line 3546‐2. (a–d) Commercially mature L‐Wty fruit (a and c) and S‐Wty fruit (b and d). (e–h) Light microscope images of L‐Wty cucumber fruit tubercules at different stages (‐7, ‐2, 0 and 13 days postanthesis (DPA)). (i–k) Images of paraffin sections of L‐Wty cucumber fruit tubercules at different stages (‐7, ‐2 and 0 DPA). (l) Semithin section of L‐Wty cucumber fruit tubercules at 0 DPA. (m–s) Light microscope images (m–p) and images of paraffin sections (q–s) of S‐Wty fruit tubercules at different stages (‐7, ‐2 and 0 DPA). (t) Semithin section of S‐Wty fruit tubercules at 0 DPA. (e, i, m, q) represent ‐7 DPA, (f, j, n, r) represent ‐2 DPA, (g, k, o, s) represent 0 DPA, respectively. Tu, tubercule; Sp, spine. When spines are combined with tubercules, fruits have a characteristic warty (Wty) trait. Scale bars: 2 cm (a, b), 200 μm (e, f, g, i, j, k, l, m, n, o, q, r, s, t) and 1 mm (h, p).

**Figure 2:**
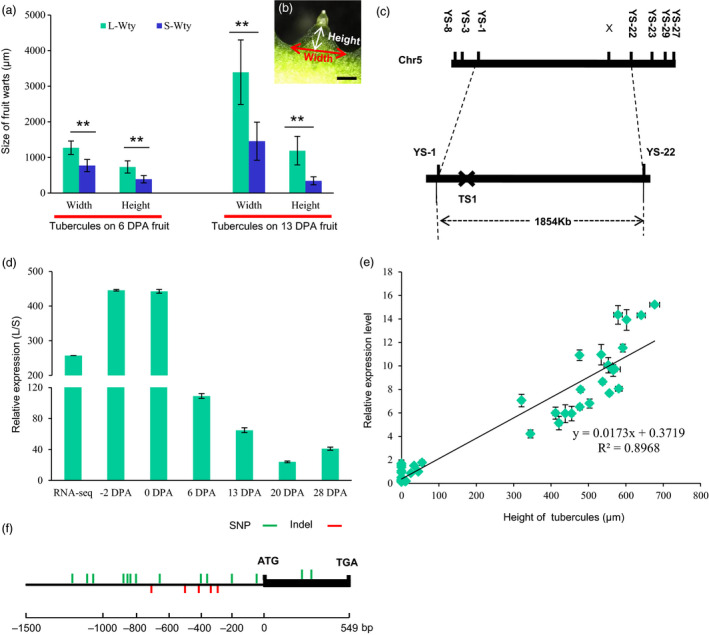
Positional cloning of the *CsTS1* gene. As shown in (b), the width and height of the tubercules on the fruits at 6 DPA and 13 DPA were analysed (a). Data are shown as means ± SD (*n *=* *20), **P <* *0.01. (c) The location of *CsTS1* on chromosome 5 between the markers YS1 and YS22. (d) The relative transcript abundance of *CsTS1* in 3546‐1 and 3546‐2 fruits at different stages. Cucumber *actin* was used as an internal control. Error bars indicate the standard deviations of three independent replicates. (e) Association analysis of *CsTS1* expression and height of tubercules on 0 DPA fruits in 44 different cucumber lines. A significant correlation between the expression levels of *CsTS1* (y) and height of tubercules (x) was found; the trend line was *y* = 0.0173*x* + 0.3719 (*R* ^2^ = 0.8968). (f) Allelic variation between 3546‐1 and 3546‐2 in the candidate gene *CsTS1*. Scale bar: 1 mm. The asterisk indicates that the fruit tubercules on 3546‐1 are significantly larger than those on 3546‐2.

We developed a small tubercule cucumber introgression line, 3546‐2, in the genetic background of 3546‐1, a North China‐type cucumber inbred line with an L‐Wty trait. The phenotype of the fruit tubercules of 3546‐1 is significantly different from that of 3546‐2. The fruit tubercules on 3546‐1 are much larger than those on 3546‐2, but there are no obvious differences between 3546‐1 and 3546‐2 in terms of fruit size and spine morphology (Figure 1a–d). Previous studies have shown that the development of fruit tubercules can be divided into three stages: the initiation stage (before 2 days before flowering [DBF]), the development stage (2 DBF‐13 days postanthesis [DPA]) and the senescence stage (after 13 DPA) (Yang *et al*., 2014). Therefore, morphological and cytological observations of fruit tubercules at ‐7, ‐2, 0 and 13 DPA in both 3546‐1 and 3546‐2 were made (Figure 1e–t). Fruit tubercules were not observed on the surface of ‐7 DPA fruit (Figure 1e,i,m,q). Fruit tubercules in 3546‐1 were observed from ‐2 DPA to 13 DPA, during which time tubercule expanded rapidly (Figure 1f–h,j–l). Although fruit tubercules in 3546‐2 emerged on the fruit at the same stage, the size of the fruit tubercules in 3546‐2 was always smaller than those in 3546‐1 (Figure 1n–p,r–t). At 6 DPA, the width and height of the fruit tubercules of 3546‐1 were approximately 1,272 ± 189 and 732 ± 171 μm, respectively, while the width and height of those from 3546‐2 were approximately 770 ± 170 and 387 ± 104 μm, respectively. Similarly, at 13 DPA, the width and height of the fruit tubercules from 3546‐1 were approximately 3,393 ± 906 and 1,189 ± 402 μm, respectively, while the width and height in those from 3546‐2 were approximately 1,456 ± 536 and 346 ± 112 μm, respectively (Figure 2a and b). To analyse the inheritance of the small tubercule phenotype in 3546‐2, genetic analysis of F_1_ and F_2_ plants derived from the cross of 3546‐1 × 3546‐2 was conducted. All F_1_ and BC_1_ plants had large‐warty fruit, and chi‐square tests were consistent with a ratio of 1 L‐Wty fruits: 1 S‐Wty fruit in BC_2_ backcross population. In the F_2_ population, cucumber plants with large and small tubercules segregated in a 3:1 ratio (477 L‐Wty vs. 167 S‐Wty; total of 624; χ^2^=0.251 <  χ_0.05,1_=3.84; *P* > 0.05; Table S1). This indicates that the small tubercule phenotype of 3546‐2 was controlled by a single recessive nuclear gene, designated as *CsTS1*.

### Map‐based cloning of *CsTS1*

Using the F_2_ population and molecular genetic markers of single nucleotide polymorphism between YS1 and YS22, *CsTS1* was mapped to a physical region of 1,853.75 kb that included 174 candidate genes (Figure 2c). RNA from the ovaries of 3546‐1 and 3546‐2 at 0 DPA was sequenced, and *Csa5G523090*, encoding a homolog of the *Arabidopsis thaliana* oleosin family protein, was the only one of 174 complete candidate genes that was differentially expressed in 3546‐1 and 3546‐2 (Figure S1 and Table S2). In our DGE data, the expression of *Csa5G523090* was over 260‐fold lower in the S‐Wty cucumber 3546‐2, and to confirm the difference in expression of *Csa5G523090* in 3546‐1 and 3546‐2, we used quantitative real‐time PCR (qRT‐PCR) to evaluate its expression in the ovaries at various stages. The data showed that the expression of *Csa5G523090* in 3546‐1 was always significantly higher than in 3546‐2 (Figure 2d). Moreover, significant positive correlation of *Csa5G523090* transcript abundance and tubercule size was observed in 44 different cucumber lines (R^2^=0.8968, Figure 2e). *Csa5G523090* was highly expressed in all of the 22 L‐Wty cucumber lines examined and showed about 10‐fold higher expression in L‐Wty lines as compared to S‐Wty lines (Figure S2 and Table S2). PCR amplification of the *Csa5G523090* CDS and promoter showed allelic variation between 3546‐1 and 3546‐2 (Figure 2f). However, the two SNPs in the CDS region identified by sequencing and sequence alignment were synonymous mutations that would not cause changes in the amino acid sequence. The promoter of *CsTS1* contained seventeen polymorphisms between 3546‐1 and 3546‐2 in the 2‐kb region before the ATG start codon (Figure 2f and Table S4). These results prompted us to consider the *Csa5G523090* as a candidate that corresponded to TS1 and regulated the fruit tubercule size.

### *CsTS1* is highly expressed in tubercules of cucumber fruits

**Figure 3:**
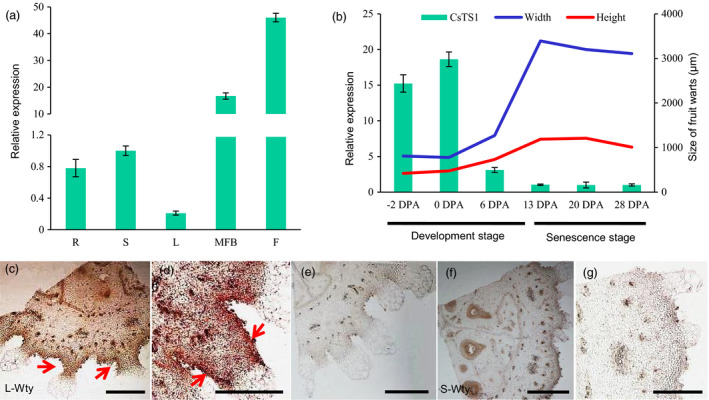
Expression pattern analysis of *CsTS1*. (a) qRT‐PCR analysis of *CsTS1* expression in different tissues (R, root; S, stem; L, leaf; MFB, male flower bud; F, fruit at 0 DPA). (b) qRT‐PCR analysis of *CsTS1* expression in peels at different stages. With the increase of the width and height of tubercules, *CsTS1* maintains a high level of expression. The cucumber gene *ACTIN* was used as an internal control. Error bars indicate the standard deviations of three independent replicates. (c and d) mRNA *in situ* hybridization of *CsTS1* in L‐Wty cucumber ovaries harvested on the flowering day. A strong signal was detected in the tubercules, epidermis and pulp adjacent to the epidermis. (f) mRNA *in situ* hybridization of *CsTS1* in S‐Wty cucumber ovaries on the day of flowering. (e and g) Negative control using the sense probe. Scale bars: 500 μm (c, e, f, g) and 200 μm (d).

To better understand the function of *CsTS1*, the expression of *CsTS1* was evaluated in various organs of 3546‐1, including roots, stems, leaves, male flower buds and fruits, using qRT‐PCR. The result showed that *CsTS1* was expressed specifically in male flower buds and fruits (Figure 3a). We also analysed the transcript levels in different developmental stages of cucumber fruits and found that the *CsTS1* transcript was more abundantly expressed in the peel at the stage of fruit tubercule development and declined rapidly as the tubercule stopped expanding (Figure 3b). In addition, the expression patterns of *CsTS1* in 3546‐1 and 3546‐2 ovaries on the day of flowering were analysed by *in situ* hybridization, and the result showed that *CsTS1* transcripts were expressed in the tubercules, epidermis and pulp adjacent to the epidermis in L‐Wty ovaries (Figure 3c and d). By contrast, no obvious signal was detected in S‐Wty cucumber tubercules, which was consistent with the qRT‐PCR results (Figure 3f). These observations confirm that *CsTS1* plays a major role in fruit tubercule development.

### *CsTS1* positively increases cell size and tubercule formation

**Figure 4:**
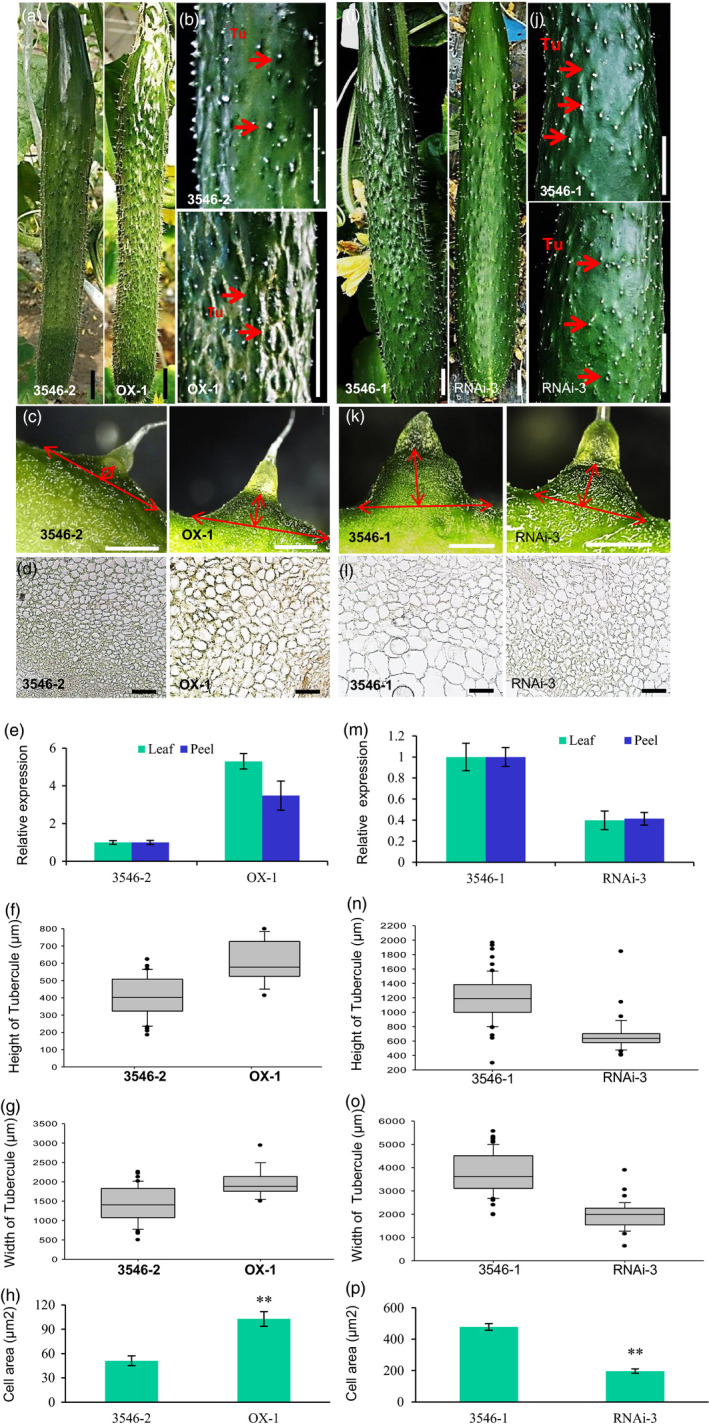
Phenotype of *35S:CsTS1* and *CsTS1‐RNAi* transgenic cucumber plants. (a–c) External morphological observations of different *35S:CsTS1* lines. (a) Whole cucumber fruits at 13 DPA. (b) and (c) Morphological observations of the fruit warts of control plant and the *35S:CsTS1*/3546‐2 transgenic cucumber plants. (d) Microscopic cross‐sections of fruit tubercules at 0 DPA. (e) qRT‐PCR analyses of *CsTS1* in leaf and peel of transgenic overexpression plants and control plants. The cucumber *ACTIN* gene was used as an internal control. Error bars indicate the standard deviations of three independent replicates. (f and g) The width and height of tubercules in control plants and *35S:CsTS1* transgenic plants. Error bars represent ±SE. (h) Quantifications of control and *35S:CsTS1* cucumber fruit tubercule cell size. (i–k) External morphological observations of *CsTS1*‐*RNAi* transgenic plants. The fruit tubercules of the RNAi line were smaller than those of control. (l) Microscopic cross‐sections of fruit tubercules at 0 DPA. (m) qRT‐PCR analyses of *CsTS1* in leaf and peel of control plants and *CsTS1*‐*RNAi* transgenic plants. The cucumber ACTIN gene was used as an internal control. Error bars indicate the standard deviations of three independent replicates. (n and o) The width and height of the tubercules in control and RNAi lines. Error bars represent ± SE. (p) Quantifications of control and *CsTS1*‐*RNAi* cucumber fruit tubercule cell size. The asterisk indicates that the cell size in the *CsTS1*‐*RNAi* line is significantly smaller than that in the control (**P < 0.01). Tu, tubercule. Scale bars: 2 cm (a, b, i, j), 1 mm (c, k) and 50um (d, l).

To clarify the function of *CsTS1* in tubercule development, the overexpression vector *35S:CsTS1* was introduced into the S‐Wty cucumber line 3546‐2. Antibiotic selection and genomic PCR were used to screen the transgenic plants (Chen *et al*., 2016; Cheng *et al*., 2015; Zhang *et al*., 2014). Notably, we found that the tubercules on the fruit surface of the transgenic cucumber lines were substantially larger than those on 3546‐2 plants (Figure 4a–c). The width and height of tubercules on fruit at 13 days postpollination (DPP) were significantly larger in transgenic plant lines than in nontransgenic 3546‐2 plants, which was caused by larger cells in transgenic fruit tubercules (Figure 4d–h). Conversely, transgenic 3546‐1 plants that underwent RNA interference (RNAi)‐mediated silencing of *CsTS1* formed smaller tubercules than those formed by nontransgenic 3546‐1 plants (Figure 4i–k). Significantly, the size of cells in the tubercules of RNAi transgenic cucumber plants was smaller, causing smaller tubercules width and height (Figure 4l–p). Thus, at current time, we have evidence that *Csa5G523090* was *TS1* affecting the fruit tubercule size in cucumber.

### The sequence changes in the *CsTS1* promoter are critical for the S‐Wty trait

As *CsTS1* is hardly expressed in S‐Wty lines*,* cloning and sequence analysis of *CsTS1* promoter region in all 44 different cucumber lines with different warts trait were performed and the results showed that *CsTS1* promoter changed in all 22 small‐warty fruit lines compared with the normal promoter in all 22 large‐warty cucumber lines (Table S3). Interestingly, the promoter sequences of all the L‐Warty lines are identical, whereas the promoter sequences of all S‐Warty lines are identical to each other (Data S1).

**Figure 5:**
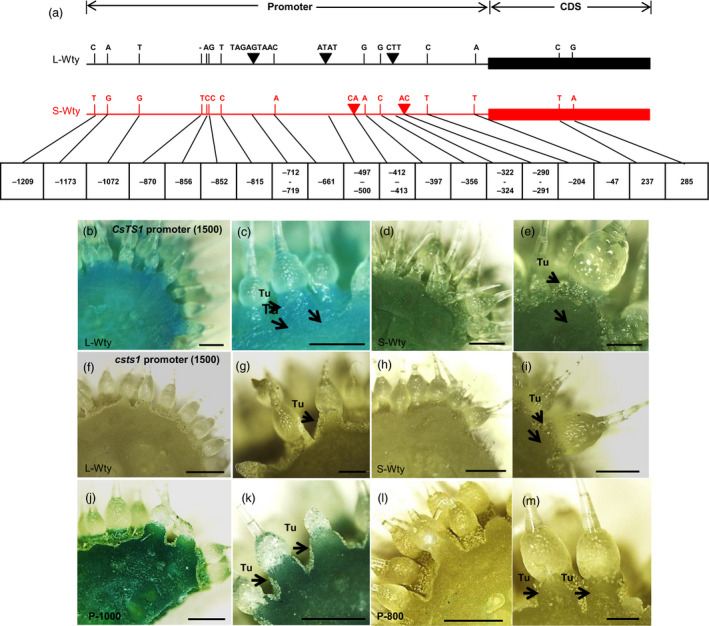
Functional analysis of the difference in the *CsTS1* promoters of 3546‐1 and 3546‐2. (a) Schematic representation of genetic variations in *CsTS1* promoter in 22 L‐Wty lines and 22 S‐Wty lines. (b–i) Functional analysis of *CsTS1* promoter activities in 3546‐1 and 3546‐2 transgenic cucumbers. GUS staining of the p*C* *sTS1*:GUS::3546‐1 (b and c) p*C* *sTS1*:GUS::3546‐2, (d and e) p*csts1*:GUS::3546‐1, (f and g) p*csts1*:GUS::3546‐2 (h and i) cucumber transgenic lines. (j–m) Deletion analysis to screen for functional cis‐elements involved in the regulation of *CsTS1* promoter activity. GUS staining of cucumber ovaries from transgenic cucumber expressing different deletions. Tu, tubercule; P‐1000, 1000‐bp promoter; P‐800, 800‐bp promoter. Scale bars: 1 mm (b‐d, h, j‐l) and 500 μm (e, g, i and m).

Therefore, we next explored whether the sequence variation in the promoter affected gene transcription using GUS staining on cucumber transgenic lines expressing *GUS* controlled by the *CsTS1* wild‐type promoters (*pCsTS1*‐GUS) and the mutant‐*CsTS1* promoter (p*csts1*‐GUS, Figure 5a). The results showed that the *CsTS1* promoter drives GUS gene expression in the tubercules, epidermis and pulp adjacent to the epidermis in both *pCsTS1::GUS/L‐Wty line 3546‐1* and *pCsTS1::GUS/S‐Wty line 3546‐2* transgenic cucumber plants, but there was no obvious expression in tubercules in the *pcsts1::GUS/L‐Wty line 3546‐1* and *pcsts1::GUS/S‐Wty line 3546‐2* transgenic cucumber plants (Figure 5b–i). The results indicate that the sequence variation in the *CsTS1* promoter is an important factor affecting gene transcription, and it influences the development of cucumber tubercules.

To investigate the functional *cis*‐elements involved in the regulation of the *CsTS1* promoter activity, progressive deletion constructs from ‐1500 to ‐200 bp in length were generated, and five promoter deletions (P‐200, P‐600, P‐800, P‐1000 and P‐1500) were fused to the GUS reporter gene. These constructs were introduced into the L‐Wty cucumber line 3546‐1, and strong GUS activity was found in the tubercules in the transgenic cucumber lines transformed with the P‐1000 promoter fragment fused to GUS (Figure 5j and k). However, the fragments smaller than 800 bp were insufficient to drive GUS expression in cucumber tubercules (Figure 5l,m and Figure S3). These results indicate that the region of the *CsTS1* promoter from ‐1000 to ‐800 bp is crucial for its preferential expression in tubercules.

To ascertain whether an epigenetic change is involved in the development of small tubercules in cucumbers, we also performed methylation analyses of the *CsTS1* promoter region in L‐Wty cucumber lines (including 3546‐1) and two S‐Wty cucumber lines (including 3546‐2) using Sequenom MassARRAY to detect the methylated CpG islands (Sutherland *et al*., 1992). Sequence analysis of *CsTS1* showed that there are many candidate CpG islands in two regions (Figure S4a). The signal pattern of the S‐Wty cucumber lines was very similar to that of the L‐Wty lines in the 2‐kb region except at the ‐852‐bp site of the promoter (Figure S4b and c). Bisulphite sequencing analysis further revealed the higher DNA methylation status was closely linked to the large tubercule trait with high gene expression of *CsTS1* (Figure S4b).

### *CsTS1* increases auxin signalling and levels in fruit tubercules

**Figure 6:**
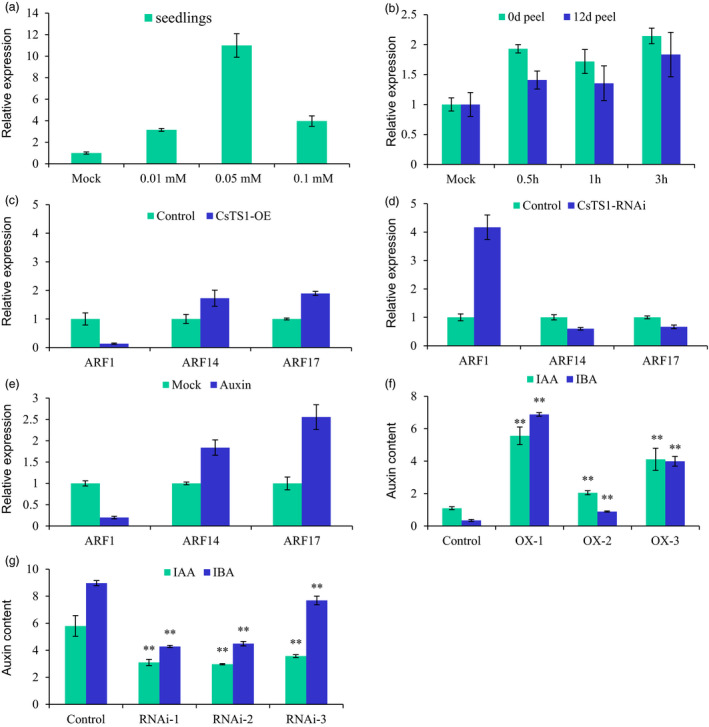
CsTS1 increases auxin signalling and levels in tubercules. (a and b) Expression profiles of *CsTS1* in response to NAA treatment. Expression pattern of *CsTS1* in the seedlings (a) and peels from cucumber fruit at 0 DPA and 12 DPA (b) after NAA treatment revealed *CsTS1* positively respond to NAA. (c and d) Expression of genes related to the auxin signalling pathway in fruit tubercules of *35S:CsTS1* and *CsTS1‐RNAi* transgenic plants. And (e) in cucumber WT tubercules treated with NAA. (f and g) Auxin contents in fruit tubercules of *35S:CsTS1* and *CsTS1‐RNAi* transgenic plants. Values are means (pg/g fresh weight) ±SD. The results are the means of three biological replicates with standard deviations. The asterisk indicates that the levels of both IAA and IBA were significantly increased in the *35S:CsTS1* tubercules and significantly reduced in the *CsTS1‐RNAi* tubercules. (Student's *t*‐test, **P < 0.01).

To better understand the regulatory mechanism of CsTS1, we screened for TS1‐interacting proteins by the yeast two‐hybrid system (Y2H) with TS1 as bait and identified 14 proteins, including three regulators of cell division and cell cycle in multicellular organism growth (homologs of SRP‐related, APC2 and BSH in Arabidopsis), two transcription factors (homologs of NAC53 and ATHB‐1 in Arabidopsis), and three important proteins in lipid metabolism pathway (ATMP2, LTP and GDSL‐motif lipase protein; Table S5). Interestingly, three of the 14 candidates are involved in auxin responses. Sequence analysis also suggested that the *cis*‐elements in the promoter of *CsTS1* included two homologous sequences of TGA element for auxin response regulator and all treatments with NAA in cucumber seedlings and peels at 0 DAP and 12 DAP induced the expression of *CsTS1* (Figure 6a and b).To investigate whether *CsTS1* influences the auxin pathway during tubercule formation, we measured the expression of three auxin signalling pathway genes, *CsARF1*,* CsARF14* and *CsARF17* in fruit tubercules of *35S:CsTS1* and *CsTS1‐RNAi* transgenic plants. The results showed that *CsARF14* and *CsARF17* showed a similar expression pattern with *CsTS1*, and *CsARF1* expression showed opposite trend in transgenic plants compared with controls (Figure 6c and d). The results that the expression of *CsARF1*,* CsARF14* and *CsARF17* were induced in response to exogenous auxin treatment confirmed their function in auxin response pathway (Figure 6e). The contents of the biologically active auxins, indole acetic acid (IAA) and indole‐3‐butyric acid (IBA) were examined in the fruit tubercules of *35S:CsTS1* and *CsTS1‐RNAi* transgenic plants. The levels of both IAA and IBA were significantly increased in the *35S:CsTS1* tubercules and significantly reduced in the *CsTS1‐RNAi* tubercules (Figure 6f and g). In addition, when the tubercules treated with NAA and antiauxins α‐(p‐chlorophenoxy) isobutyric acid (PCIB) were tested, we observed that exogenous NAA significantly promoted fruit tubercule expansion of the S‐Wty fruit line 3546‐2, while PCIB treatment resulted in smaller tubercules in the L‐Wty fruit line 3546‐1 (Figure S5). These results suggested that *CsTS1* can increase auxin signalling and levels in fruit tubercules. These results suggested that *CsTS1* might be involved in the regulation of cucumber fruit tubercule formation through influencing the content of endogenous auxin.

### The *CsTu*‐*TS1* regulatory module promotes fruit tubercule formation

**Figure 7:**
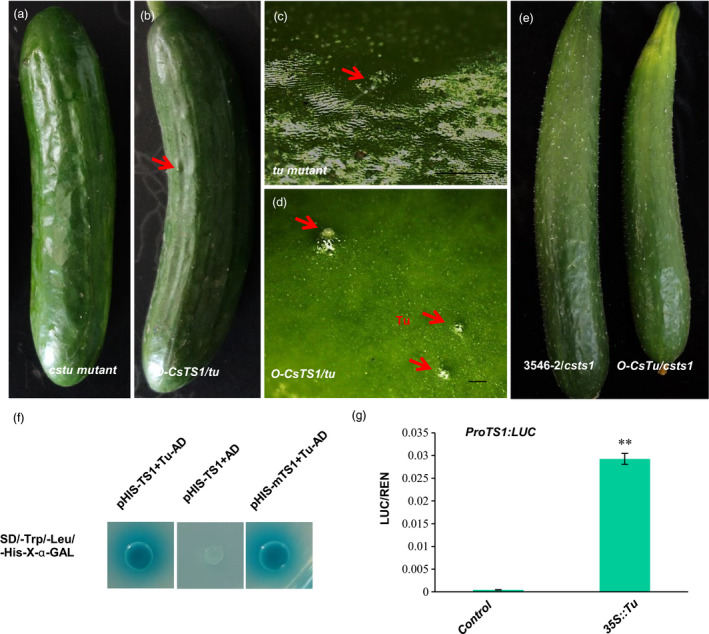
*CsTS1* interacts with tuberculate fruit gene *CsTu* to regulate tubercule formation. (a–d) Phenotype of *tu* and *35S:CsTS1::tu* transgenic cucumber plants. External morphological observations of *tu* mutant (a and c) and *35S:CsTS1::tu* (b and d). (a and b) Whole cucumber fruits at 13 DPA, (c and d) fruit warts. (e) External morphological observations of 3546‐2 and *35S:CsTu::3546‐2* transgenic cucumber fruit. (f) Yeast one‐hybrid assay and (g) a dual luciferase reporter assay showed the interactions with the CsTu and *CsTS1* 1.5‐kb promoter in *Nicotiana benthamiana* leaves. Asterisks indicate statistically significant differences (Student's *t*‐test, **P <* *0.01). Scale bars: 2 mm.

To better verify the function of *CsTu*, the expression pattern of *CsTu* was analysed in the fruits using p*CsTu*::GUS and *in situ* hybridization assays. The data showed that *CsTu* is mainly expressed in the tubercules, spines and epidermis of the fruits and this expression pattern overlaps that of *CsTS1* (Figure S6a–c). Furthermore, we constructed a *CsTu‐*RNAi vector, transformed it into 3546‐1 and obtained seven positive RNAi lines. We found that the number of tubercules on the fruit surface was significantly lower in all the RNAi lines than in the WT plants (Figure S6d–f), and the number of tubercules on the fruits at 13 DPP was 65%, 42% and 62% lower in lines 1, 2 and 3, respectively, than in WT plants (Figure S6h). Therefore, these results confirmed that *CsTu* affects the initiation of cucumber fruit tubercules. Moreover, a correlation between the expression levels of *CsTu* (y) and the expression levels of *CsTS1*(x) was found in 44 different cucumber lines (R^2^=0.562; Figure S6i). To explore the genetic relationship between *CsTS1* and *CsTu* in the fruit tubercule formation pathway, the *CsTS1* gene was overexpressed in the tubercule‐free mutant line *cstu*. There was a 4888‐bp fragment deletion from –3261 to 1627bp of the start codon in *CsTu* in the mutant *cstu*, and as a result, *CsTu* was not expressed in *cstu* compared with L‐Wty cucumber lines (Figure S7). Overexpression of *CsTS1* gene in *cstu* resulted in the emergence and development of fruit tubercules (Figure 7a‐d). However, overexpression of *CsTu* in the *csts1* mutant 3546‐2 did not cause any significant changes in the morphology of tubercules, and the small‐warty trait was not rescued (Figure 7e). Therefore, these results suggest that *CsTu* acts upstream of *CsTS1* to regulate the tubercule formation. Furthermore, a yeast one‐hybrid assay showed CsTu can bind to the *CsTS1* promoter directly (Figure 7f). Dual luciferase reporter assay confirmed this result that CsTu significantly enhanced the promoter activity of *CsTS1* in *Nicotiana benthamiana* leaves (Figure 7g). All these results suggest that CsTu functions as a positive regulator of *CsTS1* expression during the formation of fruit tubercules.

## Discussion

### CsTS1 does not regulate oil body in tubercules but is involved in the development of cucumber fruit tubercules

*CsTS1* encodes a homolog of the *Arabidopsis thaliana* oleosin proteins (Figure S1). Oleosins are plant‐specific proteins that regulate oil body size (Cabanos *et al*., 2011; Chapman and Ohlrogge, 2012; Chapman *et al*., 2012; Hsieh and Huang, 2004; Siloto *et al*., 2006; Takashi *et al*., 2008; Tzen and Huang, 1992; Tzen *et al*., 1993). In *Arabidopsis*, oleosin genes always display tissue‐specific expression, and they have been classified into three categories in accordance with their tissue‐specific expression pattern: maturing seeds, tapetum and pollen (Kim *et al*., 2002). However, in cucumber fruit, both *in situ* hybridization and *pCsTS1::GUS* assays clearly showed that *CsTS1* was expressed in the tubercules, epidermis and pulp adjacent to the epidermis (Figures 3 and 5). Furthermore, ultrastructural analysis also showed no oil bodies existed in the fruit tubercules of 3546‐1 and 3546‐2 (Figure S8). These results indicated that *CsTS1* does not regulate oil body in tubercules. However, overexpression of *CsTS1* in S‐Wty cucumber line 3546‐2 caused an increase in the size of fruit tubercules, and the size of fruit tubercules was reduced in *CsTS1‐RNAi* transgenic cucumber plants (Figure 4). *CsTS1* transcript was abundantly expressed in the fruits at the stage of fruit tubercule development and declined rapidly as the tubercule stopped expanding (Figure 3b). These results confirmed that *CsTS1* functions in regulating fruit tubercule development, unlike its homologous gene in *Arabidopsis*. We propose two explanations for the difference: (i) there are no tubercules in *Arabidopsis,* and therefore, TS1 need not be involved in tubercule development in *Arabidopsis*; (ii) repression of *AUXIN RESPONSE FACTOR10* by microRNA160 alters the expression of *OLEOSINS* in *Arabidopsis thaliana* (Liu *et al*., 2007)*,* indicating a link between auxin and *OLEOSINS*. Moreover, in our study, auxin content measurements and cytological observations in the *35S:CsTS1* and *CsTS1*‐RNAi fruit tubercules revealed that *CsTS1* can increase auxin signalling and levels in fruit tubercules. Therefore, as a homolog of *OLEOSINS*,* CsTS1* may have gained novel function in tubercule formation by regulation of auxin signalling and levels in fruit tubercules.

### The sequence changes in the promoter of *CsTS1* confer the S‐Wty trait in cucumber

DNA methylation plays critical roles in animals and plants, and the mechanisms how this DNA modification functions to regulate the genome is still unclear. However, some previous studies have shown that gene expression is affected by DNA methylation status in both animals and plants. A transgene or endogenous gene may be silenced because of DNA hypermethylation in the promoter region (Wang *et al.,* 2015b). In human, a whole‐genome comparative view of DNA methylation showed that promoter hypomethylation was positively associated with transcription in all cell types (Laurent *et al*., 2010). In *Arabidopsis*, the sites of DNA methylation were compared with microarray expression data from 79 different tissues, consistent with the results reported for human, and methylation in promoters represses gene expression (Zhang *et al*., 2006). Moreover, promoter‐unmethylated genes have significantly higher expression level than promoter‐methylated genes, indicating that promoter methylation represses gene expression in rice and maize (Banks *et al*., 1988; Brink, 1956; Das and Messing, 1994; Hollick and Chandler, 2001; Li, 2012; Li *et al*., 2014; Miura *et al*., 2009, 2010; Regulski, 2013; Wang *et al*., 2010; Zhang, 2012). The above studies confirmed that promoter methylation may be a general mechanism suppressing gene expression in eukaryotes. However, bisulphite sequencing analysis revealed the higher DNA methylation status at the ‐852‐bp site of *CsTS1* promoter was closely linked to the large tubercule trait with high gene expression of *CsTS1* (Figures S2 and S4). Alignment of the *CsTS1* promoter sequences revealed that a single nucleotide mutation (G to C) occurred at the ‐852‐bp site, causing the loss of the CG sites. The previous studies have shown that alterations in promoter sequence resulting in the modulation of gene expression can drive evolutionary changes (Wray, 2003). Deletion analysis of *CsTS1* promoter showed that the region of the *CsTS1* promoter from ‐1000 to ‐800 bp is crucial for its preferential expression in tubercules (Figure 5j–m), indicating that the allelic variation at the ‐852‐bp site of *CsTS1* promoter may influence the development of tubercule. Moreover, variation in the *CsTS1* promoter resulting in a low expression level of *CsTS1* was observed in all 22 S‐Wty cucumber lines (Figure S2). Upon transformation, only the L‐Wty‐type promoter conferred high expression of *CsTS1* (Figure 5a–h). These results mean that the sequence changes in the *CsTS1* promoter region*,* but not the decreased DNA methylation, may be the causal genetic variation for the S‐Wty trait.

### The *CsTu–TS1* regulatory module determines fruit tubercule formation in cucumber

The tuberculate fruit gene *CsTu* was cloned by map‐based cloning, and was shown to encode a C_2_H_2_ zinc finger protein, homologs of which play an important role in plant development and organ differentiation (An *et al*., 2012; Yang *et al*., 2014). Transgenic complementation of *CsTu* in nWty cucumbers line S06 demonstrated that *CsTu* is required for the warty fruit phenotype in cucumber (Yang *et al*., 2014). However, how *CsTu* works in the formation of large tubercules remains unknown. In our study, we generated transgenic *CsTu‐RNAi* cucumber plants and found that lower expression of *CsTu* significantly decreased the number of tubercules in the cucumber fruit, indicating that Tu affect the tubercule initiation (Figure S6d–f). *CsTu* is predominantly expressed in the tubercules, epidermis and pulp adjacent to the epidermis of the fruits (Figure S6a–c), and its expression overlaps with that of *CsTS1*. Furthermore, the expression level of *CsTu* and *CsTS1* in different cucumber lines showed a similar trend (Figure S6i). The results indicate that there are close relationships between *CsTu* and *CsTS1*. Molecular and genetic analyses showed that *CsTu* acts upstream of *CsTS1* to regulate tubercule formation and directly interacts with the promoter of *CsTS1* (Figure 7). The variation in the promoter of *CsTS1* does not affect the interaction between CsTu and *CsTS1* (Figure 7f), indicating that one or more additional factors should be necessary in *CsTS1* high expression proteins regulated by CsTu.

Auxin content and the expression of three auxin signalling pathway genes were both altered in the *35S:CsTS1* and *CsTS1*‐RNAi fruit tubercules, indicating *CsTS1* may play positive functions by mediating expression of auxin‐related genes in fruit tubercules (Figure 6). Sequence analysis showed that the*cis*‐elements in the promoter of *CsTS1* included 2 homologous sequences of TGA element for auxin response regulator. Treatments with NAA in wild‐type cucumber fruit peels at 0 DAP and 12 DAP could induce the expression of *CsTS1* (Figure 6a and b). Importantly, exogenous NAA treatment significantly promoted fruit tubercule expansion of the *csts1* mutant, while PCIB treatment resulted in smaller tubercules in the L‐Wty fruit line 3546‐1 (Figure S5). Taken together, these results suggested that *CsTS1* can directly increase auxin signalling and levels in fruit tubercules.

Previous studies have found that *CsTu* can up‐regulate CTK hydroxylase‐like genes, *Csa5M644580* and *Csa5M224130*, which promote CTK biosynthesis, which stimulates cell division and leads to formation of fruit tubercules (Yang *et al*., 2014). In our study, the cytokinin contents and expression level of CTK hydroxylase‐like genes, *Csa5M644580* and *Csa5M224130* in L‐Wty line 3546‐1 and S‐Wty line 3546‐2 fruit warts, were measured at 0 DPA, and there was no difference in the contents of cytokinin and the expression level of *Csa5M644580* (Figure S9a). However, the expression of CTK hydroxylase‐like genes, *Csa5M224130* in L‐Wty line 3546‐1, was less than S‐Wty line 3546‐2 (Supplemental Figure 9a). Moreover, we also measured the cytokinin contents in *35S:CsTS1* and *CsTS1‐RNAi* transgenic plants, and the levels did not changed (Supplemental Figure 9b). And the number of cells in fruit tubercules was not changed in fruit tubercules *of 35S:CsTS1* and *CsTS1‐RNAi* transgenic plants (Figure 4). Therefore, *CsTS1* is probably not involved in the pathway of cytokinin regulated by *CsTu*.

As *CsTu* encodes a C_2_H_2_ zinc finger transcription factor, it is possible that CsTu also directly regulates the expression of auxin‐related genes. The expression of auxin‐related genes was analysed in S‐Wty line 3546‐2 and *35S:CsTu::3546‐2* transgenic plants. However, the result showed that the expression level of the three auxin‐related genes unchanged in *35S:CsTu::ts1* than that in S‐Wty line 3546‐2 (Supplemental Figure 10a). In addition, we measured the auxin contents in the *35S:CsTu::csts1* transgenic fruit tubercules at 0 DPA and 12 DPA, and the levels of both IAA and IBA did not changed (Supplemental Figure 10b, c). Therefore, *CsTu* may not directly regulate the formation of auxin in cucumber tubercule.

Based on the results of this study, we speculated that CsTu regulates the expression of *CsTS*1 through directly binding to the promoter of *CsTS1,* and *CsTS1* can promote auxin biosynthesis which promotes the cell growth in cucumber tubercules. However, CsTu can also up‐regulate CTK hydroxylase‐like genes, *Csa5M644580* and *Csa5M224130*, which promote CTK biosynthesis, which stimulates cell division and leads to formation of fruit tubercules (Yang *et al*., 2014). The two regulatory pathways of *CsTu* is likely to independent in tubercule formation.

## Experimental procedures

### Plant materials and growth conditions

We developed a small tubercule cucumber introgression line, 3546‐2, in the genetic background of 3546‐1, a North China‐type cucumber inbred line with an L‐Wty trait. We used DRT4685 (Deruiter Seeds, Tianjin Derit Seeds Co. Ltd, Tianjin, China) as the donor parent and backcrossed it with 3546‐1 four times, resulting in a near isogenic line (NIL) with small tubercules. F_2_ populations of 3546‐1 and 3546‐2 were constructed to map *CsTS1*. In addition, another 42 inbred lines from different regions were used in this study. All cucumber plants were cultivated in the greenhouse under natural sunlight in Beijing, China. The modified cetyltrimethylammonium bromide (CTAB) method was used to extract genomic DNA from cucumber leaves (Murray and Thompson, 1980).

### KASPar assay

The KASPar (Kbioscience Allele‐Specific Polymorphism) platform (LGC Genomics Ltd., Hoddesdon, UK) was employed to conduct SNP genotyping in segregating populations. The SNP genotyping assay was performed as described (Wen *et al*., 2015). The KASPar SNP primers tested are listed in Table S6.

### Digital gene expression (DGE) analysis

We conducted transcriptome profiling experiments using the DGE approach. Samples were collected from cucumber fruits of 3546‐1 and 3546‐2 on the day of flowering. The DGE library construction and bioinformatics analysis of DGE data were performed as described previously (Chen *et al*., 2014). The closest homologs in *Arabidopsis* were used for GO term enrichment analysis. The up‐regulated and down‐regulated genes were analysed. We collected the corresponding closest homologs in *Arabidopsis* and used GOEAST software (Zheng and Wang, 2008) to test for GO term enrichment.

### GUS expression and staining of transgenic cucumber plants

Different fragments upstream of the start codon (ATG) of the *CsTS1* coding sequence were cloned and fused upstream of the GUS gene between the HindIII and SmaI sites in the pBI121 vector (Jefferson, 1987) to generate pCsTS1‐GUS. To make the CsTu::GUS construct, a region comprising 2000‐bp upstream of the ATG start site of the CsTu coding sequence was cloned and inserted into the pCAMBIA1391 vector (Hajdukiewicz *et al*., 1994) between the PstI and BamHI sites. Transformation and GUS assays and histochemical staining for GUS activity were performed as described previously (Jefferson, 1987; Wang *et al*., 2014). Some samples were processed for paraffin sectioning for microscopic analysis of morphology.

### Spatial and temporal expression analysis via qRT‐PCR

We extracted total RNA from the specified cucumber organs (roots, stems, female flower buds, male flower buds and fruits of different stages), using a Quick RNA isolation Kit (Huayueyang, China). The PrimeScript First Strand cDNA Synthesis Kit (Takara) and SYBR^®^ Premix Ex Taq (Takara, Dalian, China) were used to synthesize cDNA and quantitative real‐time RT‐PCR (qRT‐PCR), respectively. The cucumber *ACTIN* gene was used as an internal control (Yang *et al*., 2014). Three biological replicates and three technical replicates were performed to ensure the correctness of the experimental results. The gene‐specific qRT‐PCR primers are listed in Table S6.

### *In situ* hybridization

Cucumber ovaries harvested on the day of flowering were fixed, embedded, sectioned and hybridized with digoxigenin‐labelled probes as previously described (Zhang *et al*., 2014). Using the T7 and SP6 RNA polymerases (Roche, Shanghai, China), we obtained digoxigenin‐labelled sense and antisense RNA probes. The primer pairs used are listed in Table S6.

### Methylation analysis by Sequenom MassARRAY

Cucumber genomic DNA from ovaries harvested on the flowering day was extracted using a DNeasy Plant Mini Kit (Huayueyang, China). The DNA was then treated with bisulphite reagent using the two‐step modification procedure outlined in the Imprint DNA Modification kit (Sigma, Huayueyang, Beijing, China). Specific primer pairs were designed (Table S6). The bisulphite‐treated DNA was amplified, and the PCR products were prepared for the quantitative analysis of methylation according to the protocol provided by the manufacturer of the MassARRAY system.

### Cucumber transformation

The full‐length CsTS1 coding region was amplified and inserted in the reverse orientation into the XbaI and SmaI sites of the Pbi121 vector. The *CsTS1* overexpression constructs were transformed into the cucumber 3546‐2 line and another S‐Wty cucumber line, and a 294‐bp specific fragment of the CsTS1 CDS was used to generate the CsTS1‐RNAi construct using gene‐specific primers containing AscI (5’ end) SwaI (3’ end) sites or SpeI (5’ end) and BamHI (3’ end) sites. The recombinant plasmids were transformed into the cucumber 3546‐1 (WT) line using a cotyledon transformation method as previously described (Wang *et al*., 2014). The primers are listed in Table S6.

### Extraction and quantification of endogenous auxins

For auxin treatments, cucumber seedlings were incubated in liquid media with 0.01, 0.05 and 0.1 mm NAA. Cucumber peels at 0 DPA and 12 DPA were sprayed with 0.05 mm NAA, and peels were collected at 0.5, 1 and 3 h after treatment.

Fresh fruit tubercules (50 mg) at 0 DBF were frozen in liquid nitrogen. As previously described, the extraction and quantification of endogenous auxins and cytokinin were performed using the HPLC electrospray ionization tandem mass spectrometry (HPLC‐ESI‐MS/MS) (Wu *et al*., 2016).

For NAA and p‐chlorophenoxy isobutyric acid (PCIB) treatments, developing fruits from 3546‐1 were sprayed with 0.1 mm PCIB (auxin antagonist) from 0 DPA to 12 DPA, once a day. Developing fruits from 3546‐2 were treated with 50 μM NAA.

### Yeast one‐hybrid assay

The coding region sequences of *CsTu* were cloned into the pGADT7‐rec2 vector (Clontech). The promoter of CsTS1 in 3546‐1 and 3546‐2 was cloned into the pHIS2 vectors using the primers listed in Table S6. All recombinant constructs were separately transformed into the yeast strain Y187. Transformants were grown on SD media ‐His/‐Leu/‐Trp, but containing X‐gal to observe the colour development of yeast colonies.

### Dual luciferase transient expression assay in tobacco leaves

The promoters of *pCsTS1* (1500 bp) were cloned into the transient expression vector pGreenII 0800‐Luc (Hellens, 2005). To generate *35S:Tu* effector construct, the coding sequence (CDS) of CsTu was cloned into pGreenII 62‐SK (Hellens, 2005). Primers for all constructs are listed in Table S5. Tobacco (*Nicotiana tabacum*) leaves were used for co‐expression studies as previously described (Yotsui, 2013). No‐effector construct was used as negative control. The dual luciferase assay reagents (Promega, Beijing, China) were used to examine the firefly luciferase and renilla luciferase. Data were collected as the ratio of LUC/REN.

## Supporting information


**Figure S1** Phylogenetic analyses and protein alignment of CsTS1 and its homologues in Arabidopsis.
**Figure S2** Expression analysis of *CsTS1* in different cucumber lines.
**Figure S3** Functional analysis of *CsTS1* promoter activities.
**Figure S4** DNA Methylation analysis of the *CsTS1* region in 2 L‐Wty cucumber lines and 2 S‐Wty cucumber lines.
**Figure S5** The effect of exogenous NAA and PCIB on fruit tubercule expansion of cucumber.
**Figure S6** The expression pattern and functional analysis of *CsTu*.
**Figure S7** Analysis of the difference of *CsTu* in the nWty *tu* mutant and L‐Wty lines.
**Figure S8** Electron microscopy images of the cells in the 3546‐1 (a) and 3546‐2 (b) fruit tubercules.
**Figure S9** Expression of two CTK hydroxylase‐like genes and cytokinin contents in fruit tubercules of *35S:CsTS1* and CsTS1‐RNAi transgenic plants.
**Figure S10** Expression of three auxin signalling pathway genes and auxin contents in fruit warts of *35S:CsTu::csts1* transgenic plants.

**Table S1** Segregation analysis of the L‐Wty/S‐Wty fruit traits in the F_2_, BC_1_ and BC_2_ progenies.
**Table S2** Up‐regulated and down‐regulated genes for 3546‐1 and 3546‐2 cucumber ovaries at 0 DBF.
**Table S3** Inbred cucumber lines from different regions.
**Table S4** Allelic variations at the *CsTS1* locus.
**Table S5** The proteins interacted with CsTS1.
**Table S6** Primers used in this study.

**Data S1** cDNA and promoter sequences of CsTS1 in different cucumber inbred lines.
