# Supplementary material for: A CsTu‐ TS1 regulatory module promotes fruit tubercule formation in cucumber
Source: Plant Biotechnol J. 2018 Jul 22;17(1):289–301. doi: 10.1111/pbi.12977 (PMC6330641; doi:10.1111/pbi.12977)
Supplement: Supplementary file 1 — Figure S1 Phylogenetic analyses and protein alignment of CsTS1 and its homologues in Arabidopsis. Figure S2 Expression analysis of CsTS1 in different cucumber lines. Figure S3 Functional analysis of CsTS1 promoter activities. Figure S4 DNA Methylation analysis of the CsTS1 region in 2 L‐Wty cucumber lines and 2 S‐Wty cucumber lines. Figure S5 The effect of exogenous NAA and PCIB on fruit tubercule expansion of cucumber. Figure S6 The expression pattern and functional analysis of CsTu. Figure S7 Analysis of the difference of CsTu in the nWty tu mutant and L‐Wty lines. Figure S8 Electron microscopy images of the cells in the 3546‐1 (a) and 3546‐2 (b) fruit tubercules. Figure S9 Expression of two CTK hydroxylase‐like genes and cytokinin contents in fruit tubercules of 35S:CsTS1 and CsTS1‐RNAi transgenic plants. Figure S10 Expression of three auxin signalling pathway genes and auxin contents in fruit warts of 35S:CsTu::csts1 transgenic plants. [file PBI-17-289-s002.docx]

**Supplemental information**

**
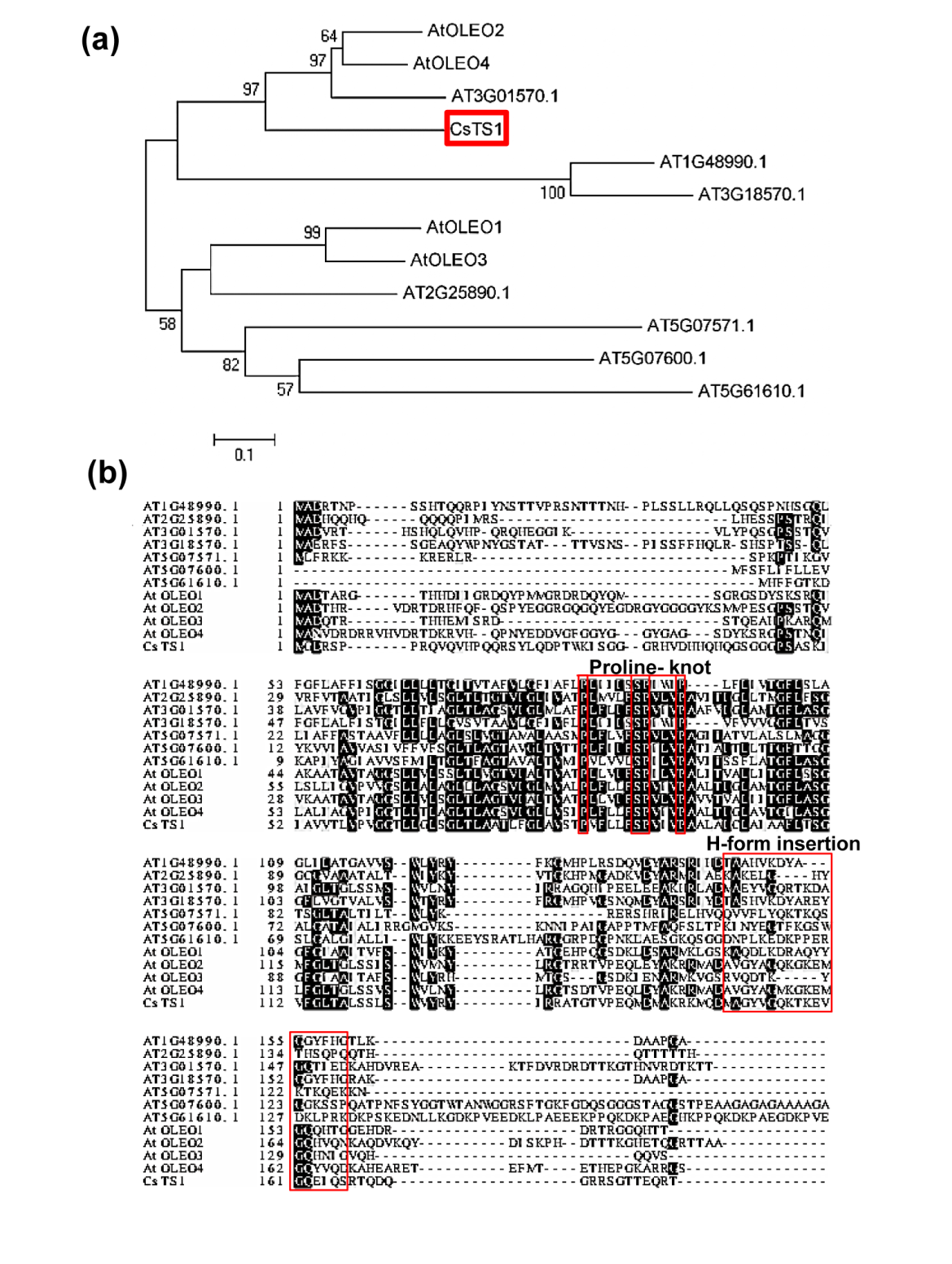
**

**Figure S1. Phylogenetic analyses and protein alignment of CsTS1 and its homologues in Arabidopsis.** (**a**) Phylogenetic tree of oleosin family proteins based on the protein sequences. (**b**) Alignment of complete amino acid sequences of CsTS1 and oleosin family proteins from *A. thaliana.* The four invariable residues in the proline knot motif are enclosed, and the location of a putative insert of an 18-residue fragment in C-terminal regions of H-oleosins is boxed.


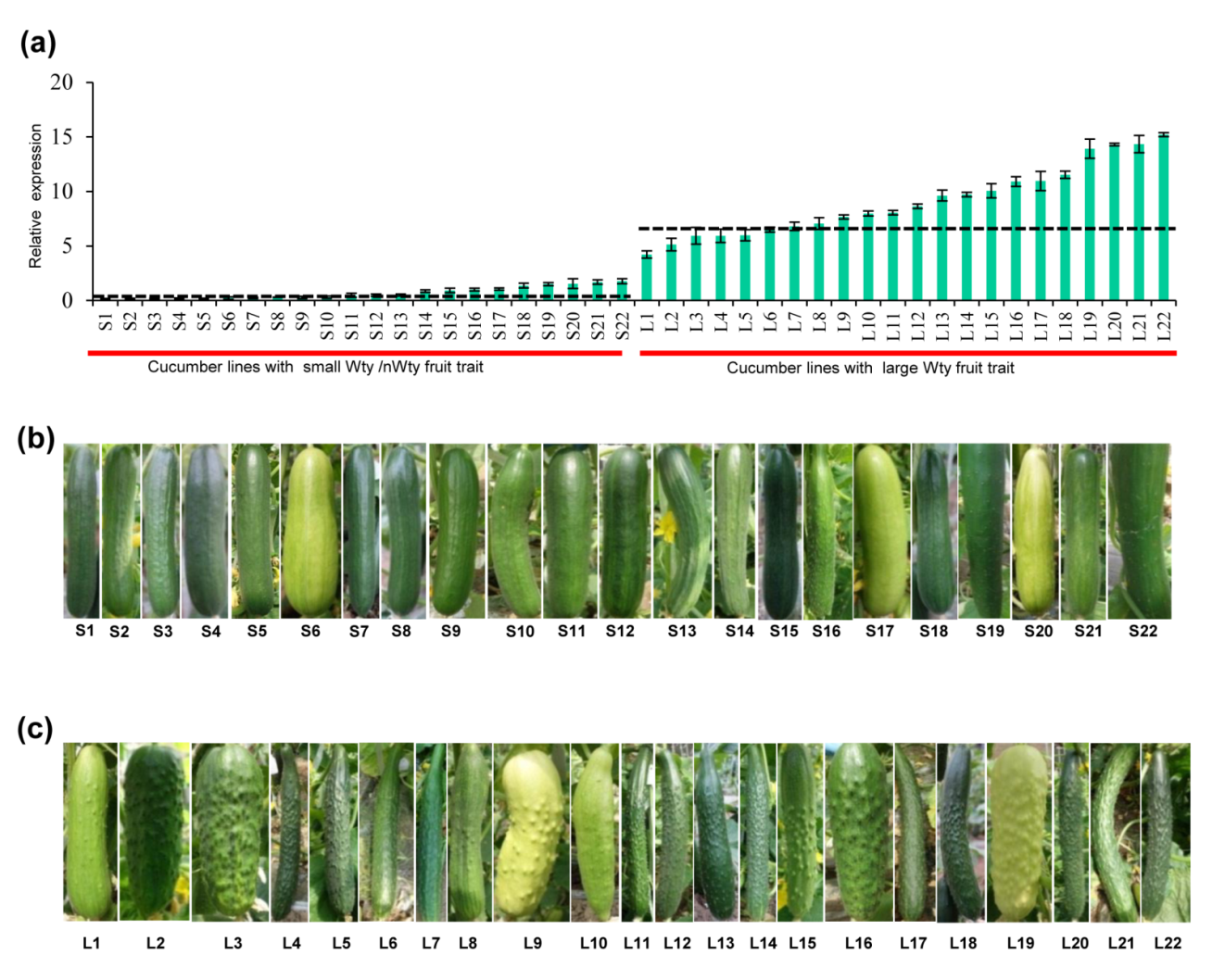


**Figure S2. Expression analysis of *CsTS1* in different cucumber lines. (a)** qRT-PCR analysis of *CsTS1* expression in 22 S-Wty or nWty cucumber lines **(b)** and 22 L-Wty cucumber lines **(c)**.

**
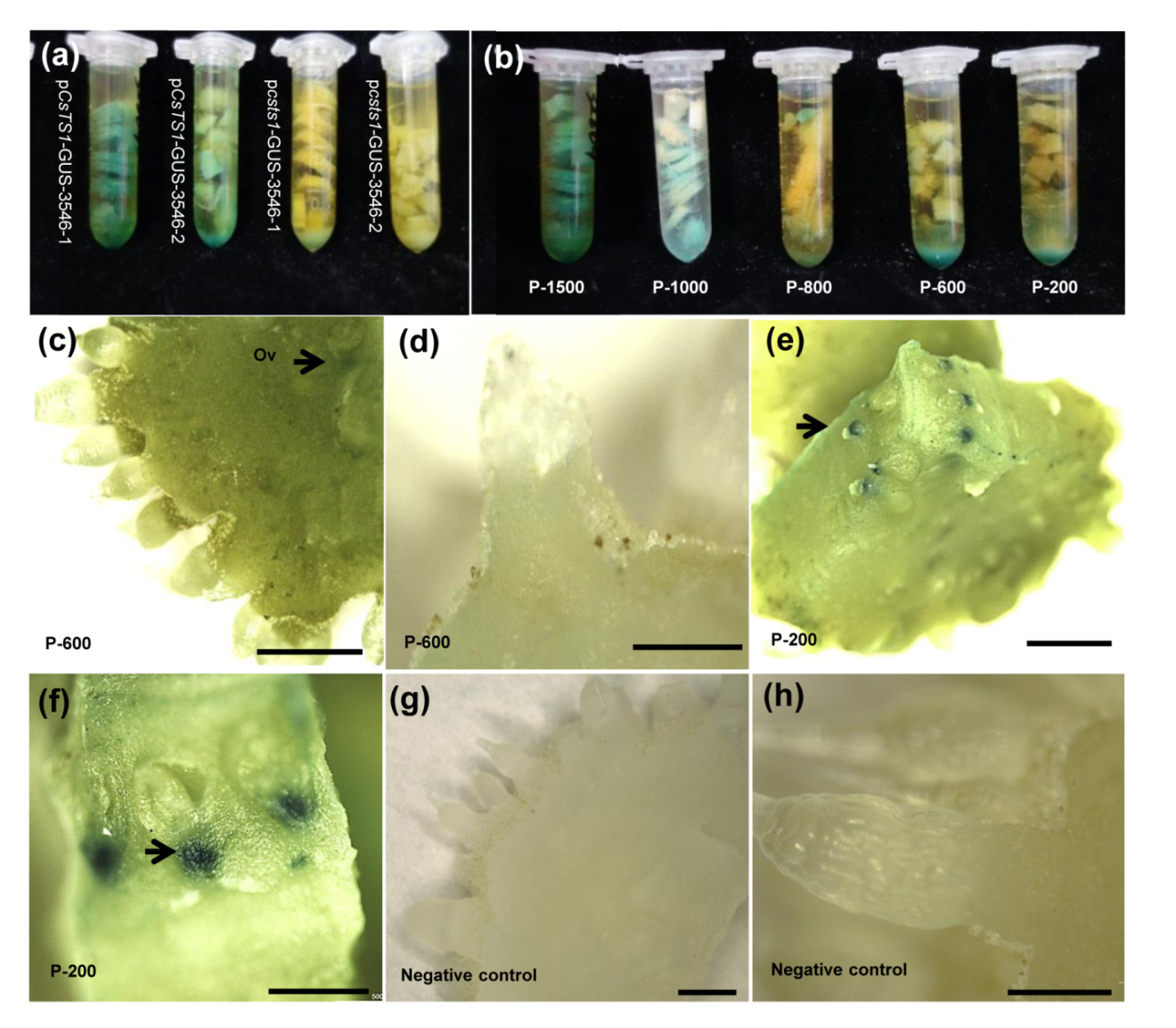
 Figure S3. Functional analysis of *CsTS1* promoter activities. (a)** GUS staining of cucumber ovaries from transgenic cucumbers with different promoters. **(b-f)** GUS staining of cucumber ovaries from transgenic cucumbers with different deletions (P-600 and P-200). **(g and h)** Negative control, GUS staining in wild-type fruit ovaries. Ov, ovules. Scale bars: 1 mm**(c, e, g)** and 500 µm **(d, f, h).**


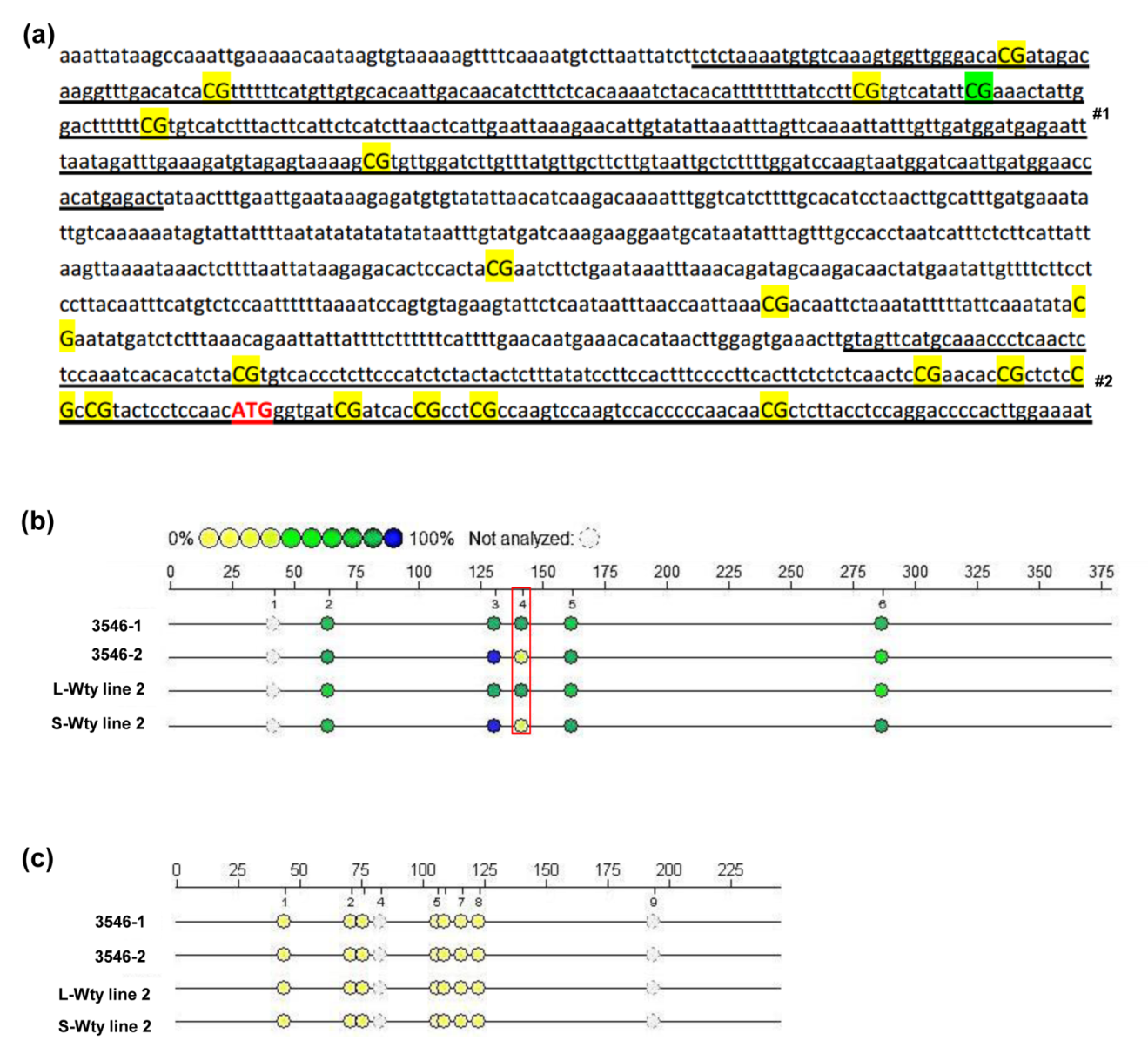


**Figure S4. DNA Methylation analysis of the *CsTS1* region in 2 L-Wty cucumber lines and 2 S-Wty cucumber lines. (a)**Analysis of the promoter and CDS region of *CsTS1*.

**(b and c)** The DNA methylation patterns upstream of region #1 and #2 in 2 L-Wty cucumber lines and 2 S-Wty cucumber lines. The green label **(a)** and the red box **(b)** indicate the regions where differences in the DNA methylation patterns of the L-Wty cucumber lines and the S-Wty cucumber lines were observed.

**
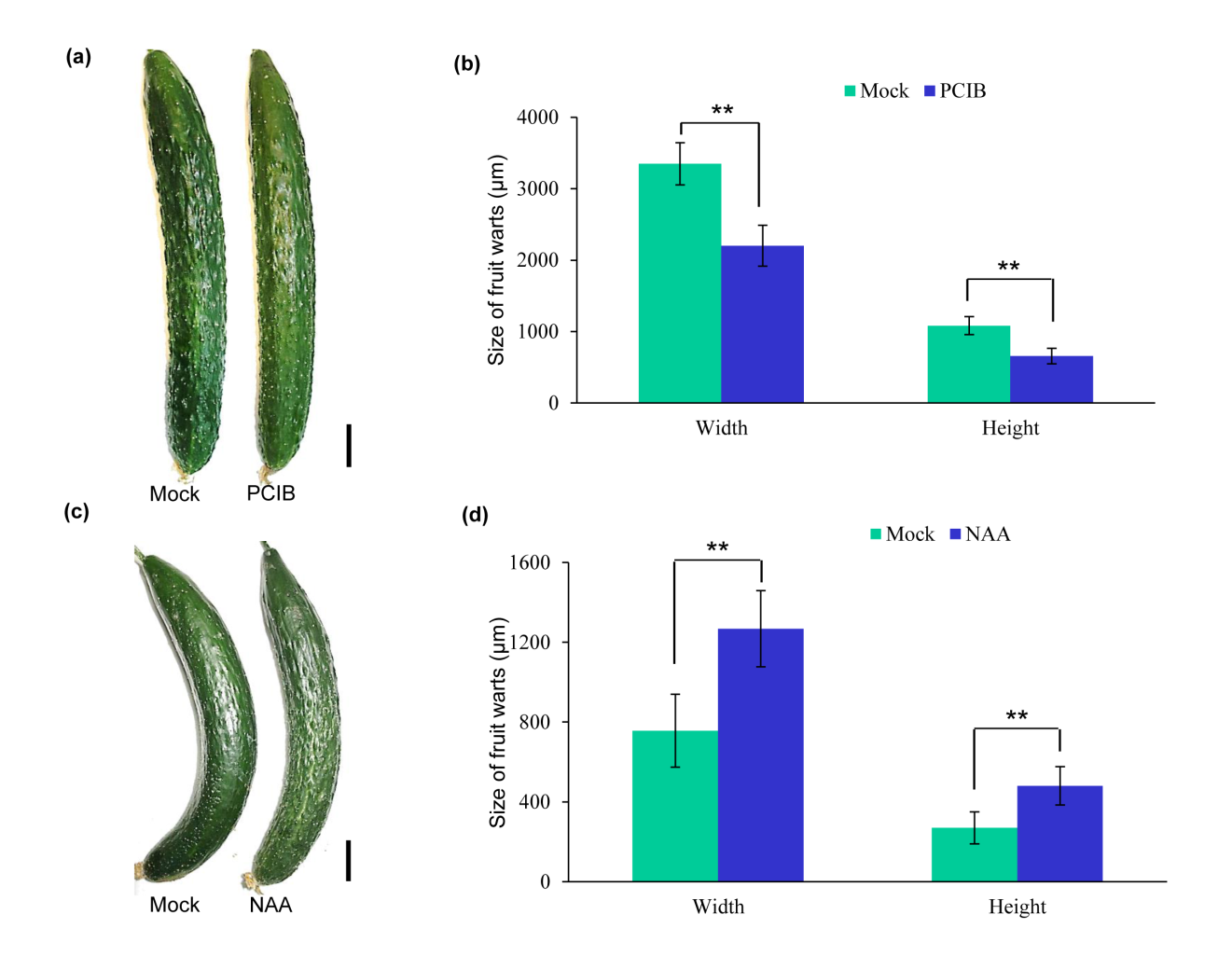
**

**Figure S5. The effect of exogenous NAA and PCIB on fruit tubercule expansion of cucumber.** Exogenous NAA significantly promoted fruit tubercule expansion of the S-Wty fruit line 3546-2 (**a** **and** **b**), while PCIB treatment resulted in smaller tubercules in the L-Wty fruit line 3546-1(**c and** **d**).

Scale bars: 2 cm **(a, c).**


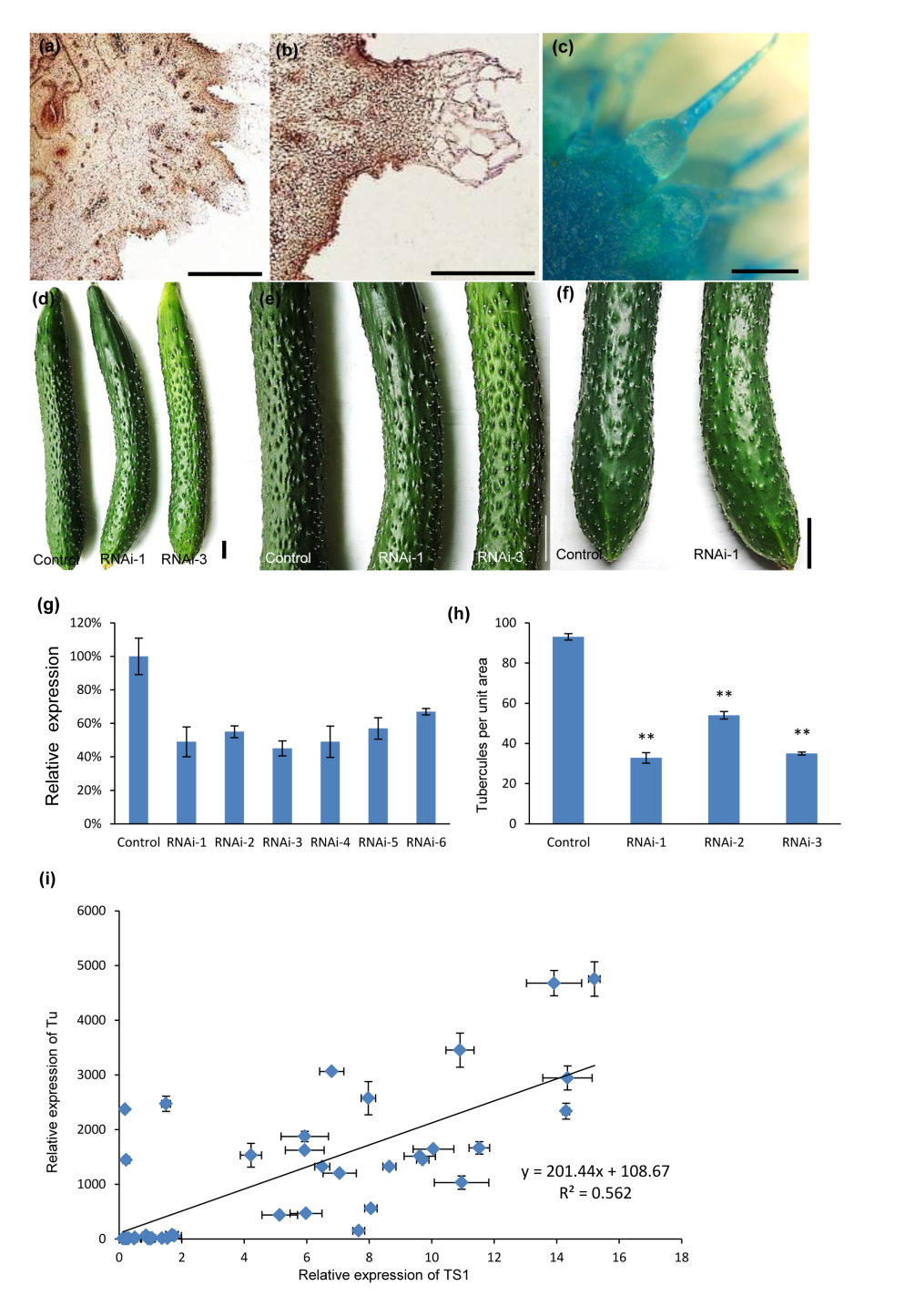


**Figure S6. The expression pattern and functional analysis of *CsTu.***

**(a and b)** mRNA *in situ* hybridization of *CsTu* in cucumber ovaries on the day of flowering.

**(c)** GUS expression (blue staining) patterns in *pCsTu-GUS* transgenic lines. GUS staining in trichome and tubercules from ovaries of the cucumber.

**(d - f)** External morphological observations of *CsTu-RNAi* transgenic plants. There were fewer fruit tubercules in the *CsTu-RNAi* line than in control.

**(g)** qRT-PCR analyses of *CsTu* in control plants and in *CsTu-RNAi* transgenic plants. The cucumber *ACTIN* gene was used as an internal control. Error bars indicate the standard deviations of three independent replicates.

**(h)** The number of tubercules in control plants and *CsTu-RNAi* lines. Error bars represent ± SE. Significant differences were determined by Student’s t test (**, P< 0.01).  **(i)** Association analysis of *CsTu* expression and *CsTS1* expression in 44 cucumber cultivars. A correlation between the expression levels of *CsTu* (y) and the expression levels of *CsTS1*(x) was found; the trend line was y=201.44x+108.67 (R^2^=0.562).

Scale bars: 500 µm **(a),** 200 µm **(b)** and 1 mm **(c)**, 2 cm (d-f)**.**


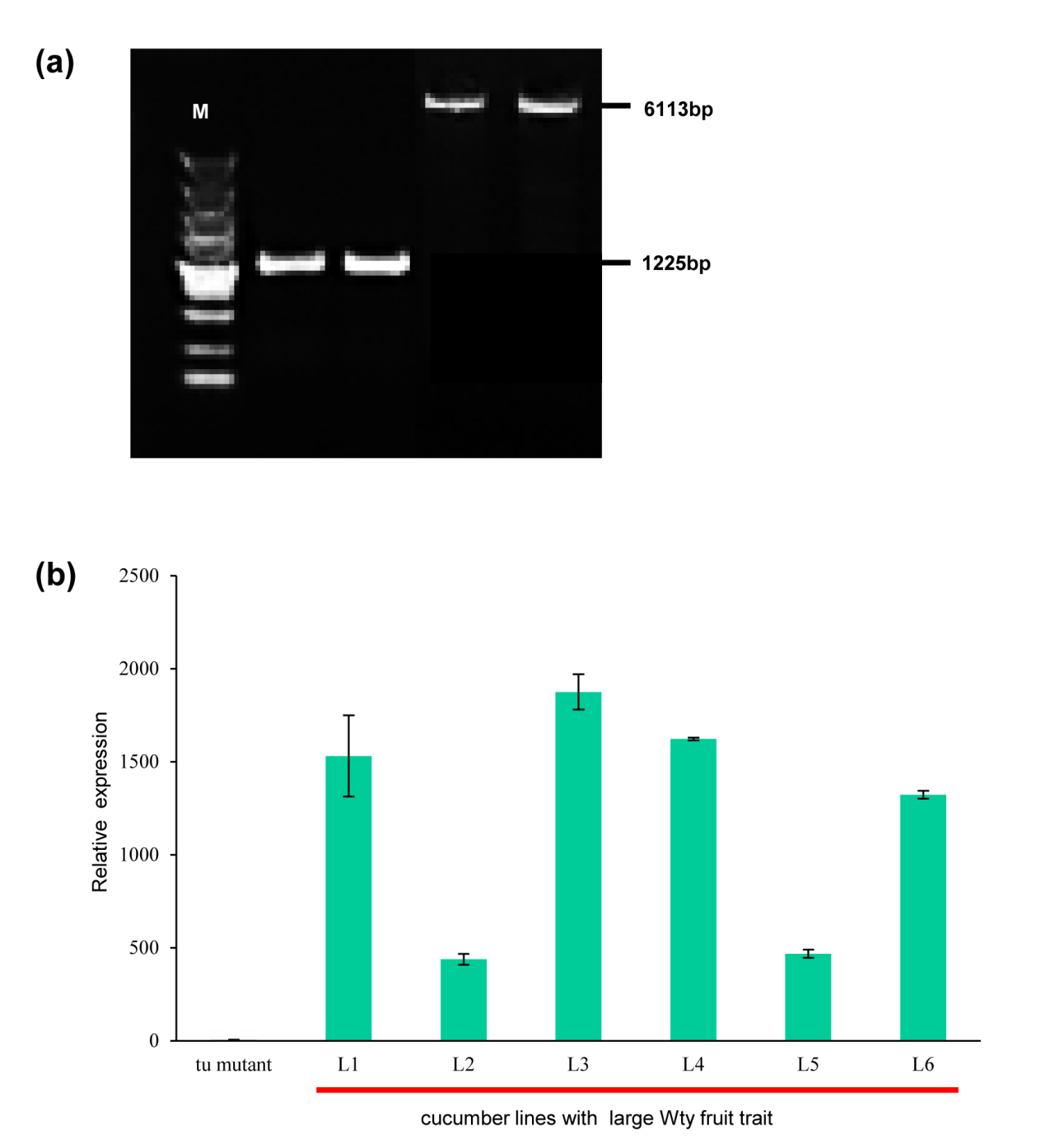


**Figure S7.** Analysis of the difference of *CsTu* in the nWty *tu* mutant and L-Wty lines.
(**a**) Confirmation of the *CsTu* gene deletion mutation in the *tu* mutant by PCR. M: Markers (DL5000).

(**b**) qRT-PCR analysis of *CsTu* expression in the nWty *tu* mutant and six L-Wty cucumber lines.


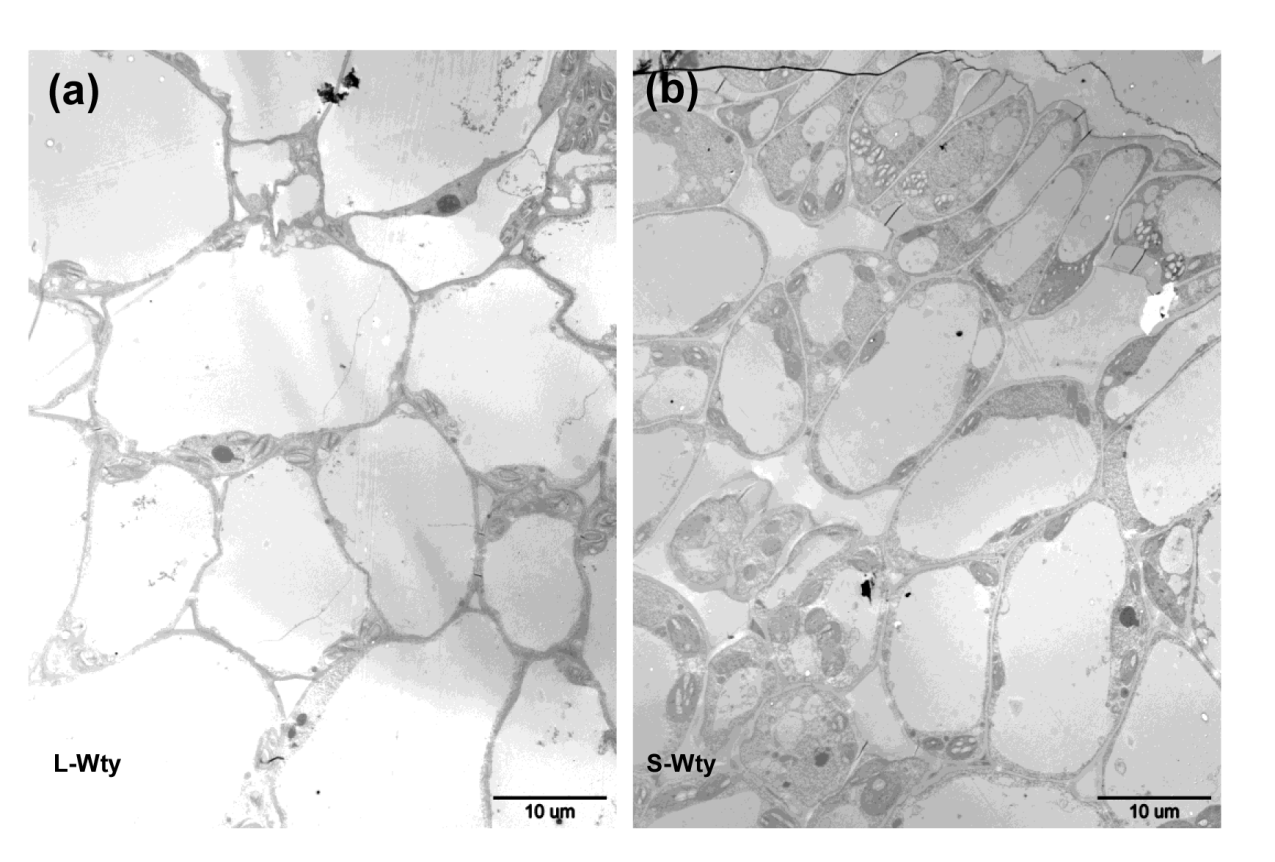


**Figure S8.** Electron microscopy images of the cells in the 3546-1 (**a**) and 3546-2 (**b**) fruit tubercules.

**
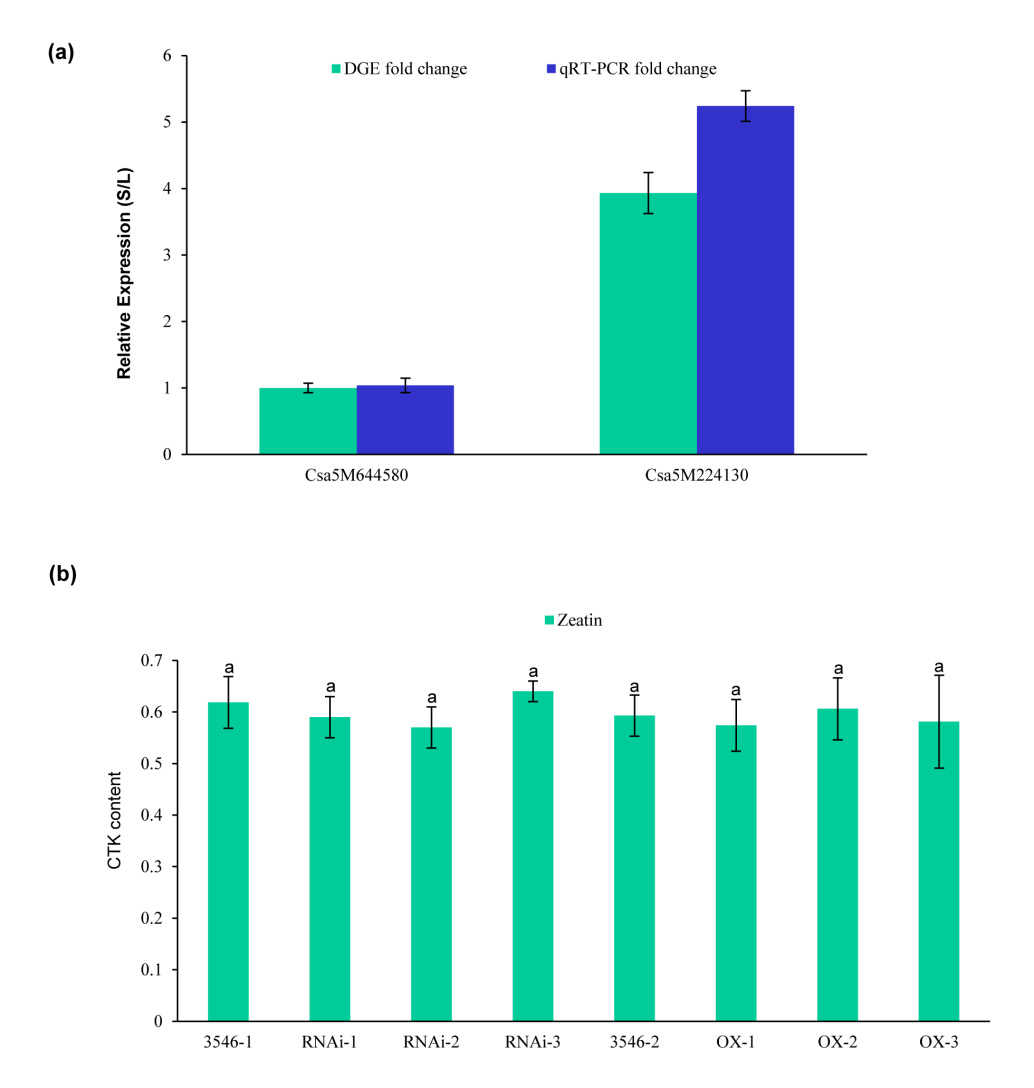
**

**Figure S9. Expression of two CTK hydroxylase-like genes and cytokinin contents in fruit tubercules of *35S:CsTS1* and CsTS1-RNAi transgenic plants.** Values are means (pg g-1 fresh weight) ±SD. The results are the means of three biological replicates with standard deviations**.** Significant differences were determined according to Duncan’s multiple range test (P < 0.05).

**
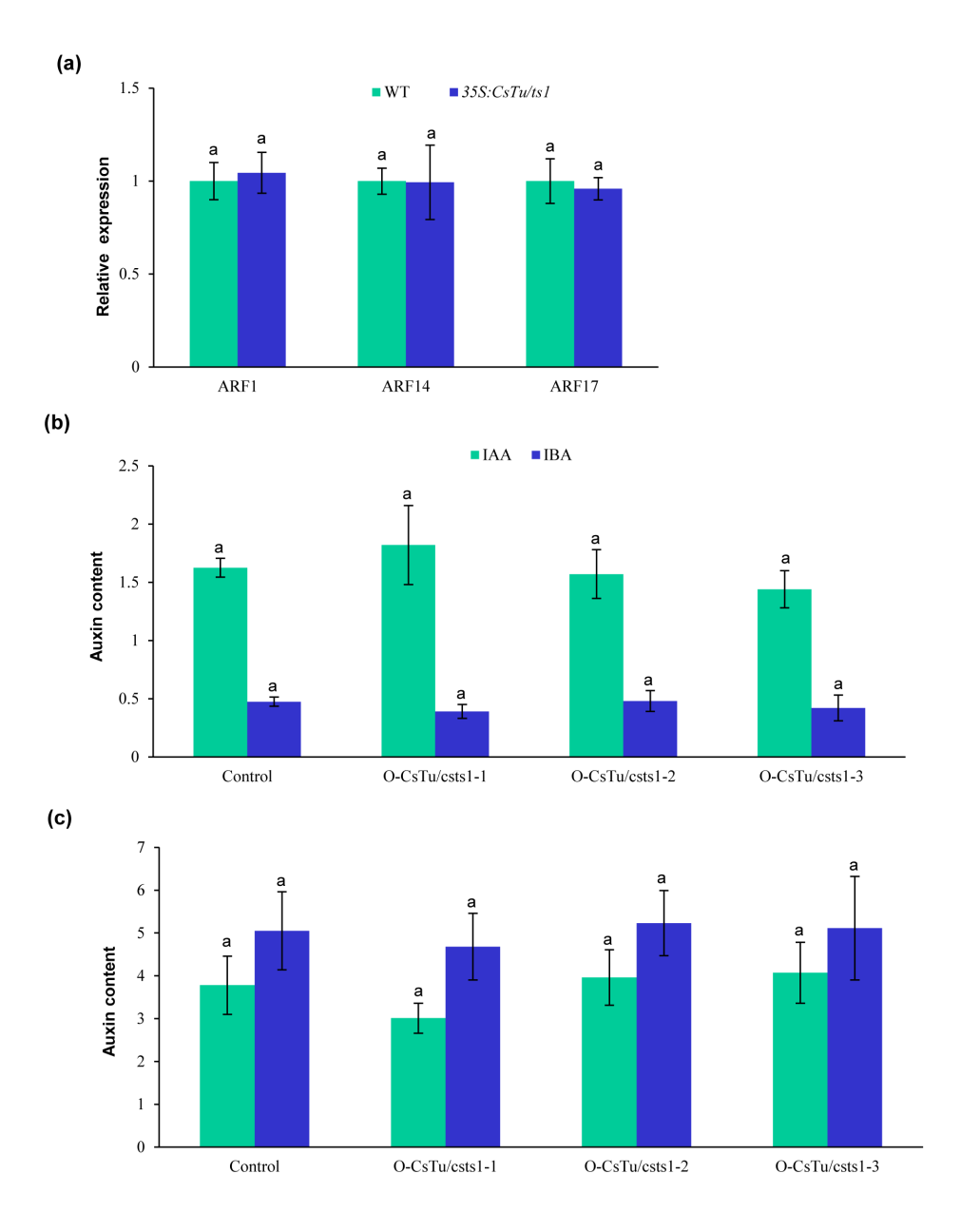
**

**Figure S10. Expression of three auxin signaling pathway genes and auxin contents in fruit warts of *35S:CsTu::csts1* transgenic plants.**

**(a)** Gene expression changes of three auxin-related genes in *35S:CsTu::ts1* plants.

**(b and c)** The contents of the biologically active auxins, indole acetic acid (IAA) and indole-3- butyric acid (IBA) in the *35S:CsTu::csts1* transgenic fruit tubercules at 0 DPA **(c)** and 12 DPA **(d)**.Values are means (pg g-1 fresh weight) ±SD. The results are the means of three biological replicates with standard deviations. Significant differences were determined according to Duncan’s multiple range test (P < 0.05).
