# Supplementary material for: A CsTu‐ TS1 regulatory module promotes fruit tubercule formation in cucumber
Source: Plant Biotechnol J. 2018 Jul 22;17(1):289–301. doi: 10.1111/pbi.12977 (PMC6330641; doi:10.1111/pbi.12977)
Supplement: Supplementary file 2 — Table S1 Segregation analysis of the L‐Wty/S‐Wty fruit traits in the F2, BC1 and BC2 progenies. Table S2 Up‐regulated and down‐regulated genes for 3546‐1 and 3546‐2 cucumber ovaries at 0 DBF. Table S3 Inbred cucumber lines from different regions. Table S4 Allelic variations at the CsTS1 locus. Table S5 The proteins interacted with CsTS1. Table S6 Primers used in this study. [file PBI-17-289-s003.docx]

**Table S1. Segregation analysis of the L-Wty/S-Wty fruit traits in the F_2_, BC_1_ and BC_2_ progenies.**

| Combinnation | Population | Total | Large-Wty fruit | Small-Wty fruit | Expected | χ^2^ value^a^ |
| --- | --- | --- | --- | --- | --- | --- |
| 3546-1×3546-2 | F_1_ | 30 | 30 |  |  |  |
|  | F_2_ | 644 | 477 | 167 | 3:1 | 0.251 |
|  | BC_1_△ | 40 | 40 | 0 |  |  |
|  | BC_2_△ | 41 | 21 | 20 | 1:1 | 0 |

**χ2 _(0.05, 1)_ = 3.84**

**Table S2. Up-regulated and down-regulated genes for 3546-1 and 3546-2 cucumber ovaries at 0 DBF.**

| **Gene ID** | **Fold Change**  **(L-Wty/S-Wty)** | **P. value** | **Annotation(TAIR10)** |
| --- | --- | --- | --- |
| **Csa5G523090**  **(*CsTS1*)** | 257.3343 | **1.35E-06** | **Oleosin family protein** |
| Csa6G504380 | 3.607752 | 4.12E-18 | BSMT1, ATBSMT1 \|S-adenosyl-L-methionine- dependent methyltransferases superfamily protein |
| Csa4G630010 | 3.088272 | 8.57E-09 | ATERF-4, ERF4, RAP2.5, ATERF4 \| ethylene responsive element binding factor 4 |
| Csa4G043950 | 2.841396 | 2.66E-06 | Pectin lyase-like superfamily protein |
| Csa4G045010 | 2.645793 | 1.03E-06 | Peroxidase superfamily protein |
| Csa5G591730 | 2.571304 | 0.00022274 | RING/U-box superfamily protein |
| Csa1G058090 | 2.32334 | 1.28E-06 | Ankyrin repeat family protein |
| Csa4G025120 | 2.241866 | 2.21E-05 | Glycine-rich protein |
| Csa5G187870 | 2.18601 | 3.33E-05 | Subtilase family protein |
| Csa3G816710 | 2.04429 | 0.00013161 | VQ motif-containing protein |
| Csa5G590070 | 1.917425 | 0.00014056 | URE \| urease |
| Csa2G171940 | 1.85405 | 7.82E-05 | Cyclopropane-fatty-acyl-phospholipid synthase |
| Csa1G168880 | 1.790621 | 0.00014172 | NAD(P)-binding Rossmann-fold superfamily protein |
| Csa1G009820 | 1.745608 | 8.43E-05 | ATEXPA1, EXP1, AT-EXP1, ATEXP1, ATHEXP ALPHA 1.2, EXPA1 \| expansin A1 \| |
| Csa4G296100 | 1.703368 | 0.00021468 | ATEXPA4, ATEXP4, expansin A4 |
| Csa1G596520 | 0.572751 | 0.0001054 | Cysteine proteinases superfamily protein |
| Csa5G158580 | 0.57037 | 0.00010857 | Copper amine oxidase family protein |
| Csa2G011500 | 0.55545 | 0.00010778 | HXXXD-type acyl-transferase family protein |
| Csa4G377740 | 0.554735 | 7.81E-05 | SHM6 \| serine hydroxymethyltransferase 6 |
| Csa4G326550 | 0.550731 | 0.0001745 | AGT3 \| alanine:glyoxylate aminotransferase 3 |
| Csa1G186610 | 0.540827 | 3.83E-05 | ATTDT, ATSDAT, TDT \| tonoplast dicarboxylate transporter |
| Csa1G172590 | 0.52766 | 0.00019911 | Heavy metal transport/detoxification superfamily protein |
| Csa3G002890 | 0.518358 | 1.02E-05 | ATMGL, MGL \| methionine gamma-lyase |
| Csa7G127380 | 0.496581 | 4.02E-05 | Atcsle1, csle1 |
| Csa4G641690 | 0.493698 | 3.16E-05 | ATMYBR1, ATMYB44, MYBR1, MYB44 \| myb domain protein r1 \| |
| Csa3G734170 | 0.488253 | 0.00022246 | Haloacid dehalogenase-like hydrolase (HAD) superfamily protein |
| Csa3G510960 | 0.486193 | 0.00022129 | HAT22 \| Homeobox-leucine zipper protein family |
| Csa5G218780 | 0.481731 | 0.00018125 | Transmembrane amino acid transporter family protein |
| Csa5G643370 | 0.465677 | 4.87E-06 | Tbl19 \| trichome birefringence-like 19 |
| Csa6G495030 | 0.458184 | 5.09E-05 | Acetamidase/Formamidase family protein |
| Csa6G511860 | 0.454547 | 0.00020914 | Ethylene-forming enzyme |
| Csa6G108440 | 0.454389 | 1.40E-05 | Cytochrome p450 78a9 |
| Csa1G183580 | 0.453697 | 7.45E-05 | Cystatin/monellin superfamily protein |
| Csa4G664500 | 0.449627 | 2.85E-05 | NAD(P)-binding Rossmann-fold superfamily protein |
| Csa1G716250 | 0.448413 | 5.64E-06 | Glycosyl hydrolases family 31 protein |
| Csa5G589940 | 0.44832 | 6.62E-06 | CCOAMT \| caffeoyl-coa 3-O-methyltransferase |
| Csa5G158590 | 0.448009 | 6.01E-06 | CYP706A6 \| cytochrome P450, family 706, subfamily A, polypeptide 6 |
| Csa6G315640 | 0.439154 | 0.000207 | Protein phosphatase 2C family protein |
| Csa5G590160 | 0.437605 | 4.75E-07 | ATMRP3, MRP3, ABCC3 \| multidrug resistance-associated protein 3 |
| Csa3G822350 | 0.433829 | 5.63E-07 | Sodium/calcium exchanger family protein |
| Csa5G643360 | 0.424872 | 3.77E-08 | Tbl19 \| trichome birefringence-like 19 |
| Csa3G132580 | 0.424754 | 4.86E-06 | Tetratricopeptide repeat (TPR)-like superfamily protein |
| Csa5G606440 | 0.424195 | 1.27E-05 | AGO7, ZIP \| Argonaute family protein |
| Csa5G139070 | 0.410086 | 1.64E-09 | LSU2 \| response to low sulfur 2 |
| Csa5G609630 | 0.40416 | 6.52E-05 | NAD(P)-binding Rossmann-fold superfamily protein |
| Csa6G513670 | 0.401758 | 1.20E-09 | APR3, PRH-26, PRH26, ATAPR3 \| APS reductase 3 |
| Csa1G062380 | 0.377487 | 2.31E-10 | APR1, APR, PRH19, ATAPR1 \| APS reductase 1 |
| Csa4G337340 | 0.371414 | 5.33E-07 | Auxin efflux carrier family protein |
| Csa3G154270 | 0.369514 | 8.39E-05 | Protein phosphatase 2C family protein |
| Csa2G034560 | 0.362035 | 5.23E-05 | KNAT2, ATK1 \| KNOTTED-like from Arabidopsis thaliana 2 |
| Csa6G490090 | 0.356754 | 7.00E-05 | ATMES1, MES1 \| methyl esterase 1 |
| Csa2G377370 | 0.349921 | 2.69E-06 | Chac-like family protein |
| Csa6G500600 | 0.337533 | 1.56E-07 | ATMIPS3, MIPS3 \| myo-inositol-1-phosphate synthase 3 |
| Csa1G051620 | 0.336085 | 6.84E-08 | Heavy metal transport/detoxification superfamily protein |
| Csa6G127480 | 0.333833 | 1.33E-05 | Protein kinase superfamily protein |
| Csa1G002810 | 0.329374 | 2.32E-10 | Peptidemethionine sulfoxide reductase 1 |
| Csa1G051870 | 0.322551 | 8.07E-05 | Disease resistance family protein / LRR family protein |
| Csa1G422480 | 0.322171 | 2.60E-08 | Xyloglucan endotransglucosylase/hydrolase family protein |
| Csa2G293360 | 0.318287 | 4.03E-05 | PLP4, PLA V, atplaivc \| PATATIN-like protein 4 |
| Csa6G395130 | 0.316659 | 3.64E-05 | Thiamin diphosphate-binding fold (THDP-binding) superfamily protein |
| Csa7G195260 | 0.314755 | 1.02E-05 | Aldolase superfamily protein |
| Csa1G699560 | 0.299972 | 1.31E-06 | Alternative oxidase family protein |
| Csa7G343870 | 0.290277 | 1.18E-13 | ATPME3, PME3 \| pectin methylesterase 3 |
| Csa7G129380 | 0.2788 | 3.91E-11 | ATCSLE1, CSLE1 \| cellulose synthase like E1 |
| Csa6G153490 | 0.262084 | 1.13E-09 | Unknown protein; |
| Csa5G224130 | 0.254317 | 1.07E-07 | CYP714A1 \| cytochrome P450, family 714 |
| Csa6G490080 | 0.249066 | 0.00014851 | ATMES1, MES1 \| methyl esterase 1 |
| Csa1G073050 | 0.234995 | 0.00010822 | Scpl31 \| serine carboxypeptidase-like 31 \| |
| Csa3G097540 | 0.190083 | 4.73E-08 | TPS21 \| terpene synthase 21 |
| Csa1G103250 | 0.18277 | 3.28E-09 | SAUR-like auxin-responsive protein family |
| Csa2G353460 | 0.167276 | 6.87E-06 | 1-amino-cyclopropane-1-carboxylate synthase 8 Encodes an auxin inducible ACC synthase |
| Csa6G518000 | 0.167021 | 6.45E-16 | Dormancy-associated protein-like 1 |
| Csa2G200420 | 0.146442 | 2.92E-05 | Indole-3-acetic acid inducible 14 |
| Csa3G874380 | 0.14319 | 3.17E-10 | DC1 domain-containing protein |
| Csa6G367080 | 0.14074 | 1.56E-18 | K-box region and MADS-box transcription factor family protein |
| Csa2G023940 | 0.139981 | 1.23E-07 | Lipoxygenase 1 |
| Csa2G024440 | 0.138696 | 5.39E-09 | Lipoxygenase 1 |
| Csa2G430570 | 0.123681 | 1.56E-10 | HXXXD-type acyl-transferase family protein |
| Csa1G051720 | 0.122716 | 7.25E-05 | Homolog of Medicago truncatula MTN3 |
| Csa4G641760 | 0.107596 | 0.00012752 | Cytochrome P450, family 81, subfamily D, polypeptide 8 |
| Csa2G027440 | 0.106306 | 5.19E-10 | Lipoxygenase 1 |
| Csa3G851720 | 0.103607 | 7.88E-06 | Dynein light chain type 1 family protein |
| Csa2G238880 | 0.094667 | 8.57E-15 | GLB1, AHB1, ARATH GLB1, NSHB1, ATGLB1, HB1 \| hemoglobin 1 |
| Csa3G180440 | 0.046404 | 8.92E-29 | PEBP (phosphatidylethanolamine-binding protein) family protein |
| Csa6G448740 | 0.034559 | 1.64E-16 | L-O-methylthreonine resistant 1 |

**Table S3. Inbred cucumber lines from different regions.**

| **Lines** | Material  number | Origins | LW/SW | p*CsTS1*  LW/SW | **Lines** | Material number | Origins | LW/  SW | p*CsTS1*  LW/SW |
| --- | --- | --- | --- | --- | --- | --- | --- | --- | --- |
| **S1** | 2336-7 | European | S | S | **L1** | 6102-13 | China | L | L |
| **S2** | 2334-4 | European | S | S | **L2** | 6102-16 | European | L | L |
| **S3** | 2336-5 | European | S | S | **L3** | 6102-14 | European | L | L |
| **S4** | 2076-2 | European | S | S | **L4** | 1567-4-3 | China | L | L |
| **S5** | 2326-2 | European | S | S | **L5** | 3544-2 | China | L | L |
| **S6** | 6101-7 | European | S | S | **L6** | **3546-1** | China | L | L |
| **S7** | 2336-8 | European | S | S | **L7** | 3461 | China | L | L |
| **S8** | 2336-2 | European | S | S | **L8** | 6101-12 | European | L | L |
| **S9** | 2319-1 | European | S | S | **L9** | 6102-18 | China | L | L |
| **S10** | 2073-2 | European | S | S | **L10** | 6101-11 | China | L | L |
| **S11** | 6101-4 | European | S | S | **L11** | 5001-1 | China | L | L |
| **S12** | 6101-6 | European | S | S | **L12** | 3546-6 | China | L | L |
| **S13** | 2321-7 | European | S | S | **L13** | 1557-8 | China | L | L |
| **S14** | 2604-8 | European | S | S | **L14** | 3542-1 | China | L | L |
| **S15** | 2077-3 | European | S | S | **L15** | 2311 | Japan | L | L |
| **S16** | **3546-2** | European | S | S | **L16** | 6102-7 | European | L | L |
| **S17** | 6103-18 | European | S | S | **L17** | 1554-2-1 | China | L | L |
| **S18** | 2076-5 | European | S | S | **L18** | 1556-13 | China | L | L |
| **S19** | 3312 | USA | S | S | **L19** | 6102-9 | China | L | L |
| **S20** | 6103-8 | European | S | S | **L20** | 3546-9 | China | L | L |
| **S21** | 2320-1 | European | S | S | **L21** | 3407 | China | L | L |
| **S22** | 3413 | China | S | S | **L22** | 3545-2 | China | L | L |

LW, Large-warty; SW, Small-warty; L, Large-Wty fruit line; S, small-Wty fruit line;

**Table S4. Allelic variations at the *CsTS1* locus.**

| **Position** | **3546-1** | **3546-2** |
| --- | --- | --- |
| **237** | **C** | **T** |
| **285** | **A** | **A** |
| **-47** | **A** | **T** |
| **-204** | **C** | **T** |
| **-290~ -291** | **--** | **AC** |
| **-322~ -324** | **CTT** | **---** |
| **-356** | **G** | **C** |
| **-397** | **G** | **A** |
| **-412~ -413** | **--** | **CA** |
| **-497~ -500** | **ATAT** | **----** |
| **-661** | **C** | **A** |
| **-712~-719** | **TAGAGTAA** | **--------** |
| **-815** | **T** | **C** |
| **-852** | **G** | **C** |
| **-856** | **A** | **C** |
| **-870** | **-** | **T** |
| **-1072** | **T** | **G** |
| **-1173** | **A** | **G** |
| **-1209** | **C** | **T** |

**Table S5. The proteins interacted with CsTS1.**

| **Gene ID** | **GO** |
| --- | --- |
| **Csa1M627500.1** | Signal recognition particle-related / SRP-related, positive regulation of multicellular organism growth |
| **Csa5M470570.1** | APC2; ubiquitin protein ligase binding / ubiquitin-protein ligase, cell division, cell cycle |
| **Csa3M171220.1** | BSH (BUSHY GROWTH); chromatin binding / protein binding, cell differentiation，auxin mediated signaling pathway |
| **Csa5M622470.1** | Arabidopsis NAC domain containing protein 53, fruit dehiscence, response to auxin stimulus |
| **Csa1M045550.1** | ATHB-1 (ARABIDOPSIS THALIANA HOMEOBOX 1); transcription factor, negative regulation of cell growth, response to auxin stimulus |
| **Csa3M816160.1** | ASD1 (ALPHA-L-ARABINOFURANOSIDASE 1), acting on glycosyl bonds |
| **Csa3M875430.1** | DRT112, copper ion binding / electron carrier, response to copper ion |
| **Csa5M139910.1** | Transport |
| **Csa1M025830.1** | tRNA pseudouridine synthase family protein |
| **Csa3M915110.1** | ATMP2; heme binding, lipid biosynthetic process |
| **Csa1M076600.1** | GDSL-motif lipase/hydrolase family protein, lipid catabolic process |
| **CsaUNM028560.1** | Protease inhibitor/seed storage/lipid transfer protein (LTP) family protein |
|  |  |

**Table S6. Primers used in this study.**

**Primers for cloning***CsTS1-F* 5'- ATGGGTGATCGATCACCGCC -3'
*CsTS1-R* 5'- TTAAGTTCTTTGTTCTGTTGTG -3'

*CsTU-F* 5'- ATGGCAGCTCTAGAAAACCA -3'
*CsTU-R* 5'- TCATGGGGCGGCCGGAGCGA -3'
**Primers for qRT-PCR***q-CsTS1-F* 5'- GCCATGTAGACCACCACCAA -3'
*q-CsTS1-R* 5'- AGAGAAGGAACACAGGCGTC -3'
*CsTU-F* 5'- ATCAAGACGATTCCACCCCC -3'
*CsTU-R* 5'- ACGCTCTTTCTTGTGGGCAT -3

*Actin-F 5'-TCGTGCTGGATTCTGGTG-3'*

*Actin-R 5'-GGCAGTGGTGGTGAACAT-3'*

**Primers for *in situ* probes***CsTS1-SP6* 5'- GATTTAGGTGACACTATAGAATGCTAATCATTGCCGTCGTCACCC-3'
*CsTS1-T7* 5'- TGTAATACGACTCACTATAGGGTCCTGCATCTTCCTCTTAGCC -3'

*CsTU-F* 5'- GATTTAGGTGACACTATAGAATGCTGCCCACAAGAAAGAGCGTC -3'
*CsTU-R* 5'- TGTAATACGACTCACTATAGGGCTCAGCGACATCGGAGCATC -3
**Primers for GUS construct***ProCsTS1 (1500)-F* 5'- CCCAAGCTTTAAGATTCTTTTACTCATCGAATGG -3'

*ProCsTS1 (1200)-F* 5'- CCCAAGCTTCCGGGTAGAATAACACATACATTTT -3'

*ProCsTS1 (1000)-F* 5'- CCCAAGCTTAAATGTCTTAATTATCTTCTCTAAAATGTG -3'

*ProCsTS1 (800)-F* 5'- CCCAAGCTTATTGAATTAAAGAACATTGTATATT-3'

*ProCsTS1 (600)-F* 5'- CCCAAGCTTATTGAATTAAAGAACATTGTATATTAAATTT -3'

*ProCsTS1 (200)-F* 5'- CCCAAGCTTATTATTATTTTCTTTTTTCATTTTGAA -3'
*ProCsTS1-R* 5'- TCCCCCGGGGTTGGAGGAGTACGGCGG -3'

*CsTU-F* 5'- AACTGCAGTAGTGTTAATCTCGTTTTACTTTTATTTATC -3'
*CsTU-R* 5'- CGGGATCCGGAAAAATGGAAGATGATAAGAGAG -3
**Primers for *CsTS1* overexpression construct***O-CsTS1* 5'- GCTCTAGAATGGGTGATCGATCACCGCC -3'
*O-CsTS1* 5'- TCCCCCGGGTTAAGTTCTTTGTTCTGTTGTG -3'

**Primers for *CsTS1-RNAi* construct***I-CsTS1-F1* 5'- AGGCGCGCCAATCATTGCCGTCGTCACCC -3'
*I-CsTS1-R1* 5'- ATTTAAATTCCTGCATCTTCCTCTTAGCC -3'
*I-CsTS1-F2* 5'- GACTAGTAATCATTGCCGTCGTCACCC -3'
*I-CsTS1-R2* 5'- CGGGATCCTCCTGCATCTTCCTCTTAGCC -3'

*I-CsTU-F1* 5'- AGGCGCGCCGCCCACAAGAAAGAGCGTCA -3'
*I-CsTU-R1* 5'- ATTTAAATCTCAGCGACATCGGAGCATC -3'
*I-CsTU-F2* 5'- GACTAGTGCCCACAAGAAAGAGCGTCA -3'
*I-CsTU-R2* 5'- CGGGATCCCTCAGCGACATCGGAGCATC -3'
**Primers for PCR identification***35S-F* 5'- TGGTTAGAGAGGCTTACGCAGCAGGTC -3'
*35S-R* 5'- GCGATCCAGACTGAATGCC -3'

**Primers for mapping of TS1 on Chr. 5**

YS-1Y 5’- GAAGGTGACCAAGTTCATGCTTTTTTTTTATCCTTCGTGTCATATTCG-3’

YS-1T 5’- GAAGGTCGGAGTCAACGGATTTTTTTTTTATCCTTCGTGTCATATTCC-3’

YS-1R 5’- GAAGTAAAGATGACACGAAAAAAGTCCAATAG-3’

YS-3Y 5’- GAAGGTGACCAAGTTCATGCTATCATGATCACCTACCTAGTATTTAATATTCTATG-3’

YS-3T 5’- GAAGGTCGGAGTCAACGGATTCTATCATGATCACCTACCTAGTATTTAATATTCTATA-3’

YS-3R 5’- GAGATAACCTATCTGACCATACAACATTTAAGTG-3’

YS-8Y 5’- GAAGGTGACCAAGTTCATGCTAATTAGAATCATCACAAGTTGTTCTAGTGAC-3’

YS-8T 5’- GAAGGTCGGAGTCAACGGATTAATTAGAATCATCACAAGTTGTTCTAGTGAT-3’

YS-8R 5’- GACTTTCTTAGCTCTCCATTAAAGTCATCCA-3’

YS-22Y 5’- GAAGGTGACCAAGTTCATGCTAAAATGCAAAATATTTCAAAGGTTCAG-3’

YS-22T 5’- GAAGGTCGGAGTCAACGGATTAAAATGCAAAATATTTCAAAGGTTCAA-3’

YS-22R 5’- CTCTCCACAAGCAAGACAAATCAACTCT-3’

YS-23Y 5’- GAAGGTGACCAAGTTCATGCTAAAAGTGTCCATACTATTATAGACAATTCTCTTTA -3’

YS-23T 5’- GAAGGTCGGAGTCAACGGATTAAAGTGTCCATACTATTATAGACAATTCTCTTTC-3’

YS-23R 5’- AATGTGATCATAGTCTTATATTGACTAGGTAAGAGAAAAA-3’

YS-29Y 5’- GAAGGTGACCAAGTTCATGCTTCTAGCCCGTGACAAAGCCAAT-3’

YS-29T 5’- GAAGGTCGGAGTCAACGGATTCTAGCCCGTGACAAAGCCAAA-3’

YS-29R 5’- GAAAACTACAATTCAAGAACATATGTTGGGAT-3’

YS-27Y 5’- GAAGGTGACCAAGTTCATGCTGATGTAGAGCAAATAAAATGGAGTAATTTTT-3’

YS-27T 5’- GAAGGTCGGAGTCAACGGATTGATGTAGAGCAAATAAAATGGAGTAATTTTC-3’

YS-27R 5’- ATCAATTCATTTGTACATATTACTATTAACTTAATCTCCA-3’

**Primers for Methylation Analysis**

**#1-** F 5’- AGGAAGAGAGTTTTTAAAATGTGTTAAAGTGGTTGG -3’

**#1-** R 5’- CAGTAATACGACTCACTATAGGGAGAAGGCTAATCTCATATAATTCCATCAATTAATCC -3’

**#2-** F 5’- AGGAAGAGAGATATTTTTTAAGTGGGGTTTTGGAG -3’

**#2-** R 5’- CAGTAATACGACTCACTATAGGGAGAAGGCTATAATTCATACAAACCCTCAACTCTC -3’

**Primers for yeast one-hybrid construct**
*TS1*-Phis2 **-** F 5’- CGAGCTCGTTGGAGGAGTACGGCGG-3’

*TS1*-Phis2 **-** R 5’-GGAATTCTAAGATTCTTTTACTCATCGAATGG-3’

*TU-AD-F* 5’- GGAATTCCATATGATGGCAGCTCTAGAAAACCA-3’

*TU-AD-R* 5’- GGAATTCTCATGGGGCGGCCGGAGCGA-3’

**Primers for dual-luciferase transient expression assay**

*TS1*-LUC **-** F -5’-TCCCCCGGGAACTATAACTGATAGACTTGTATCGCTGAT-3’

*TS1*-LUC **-** A5’- GACTAGTGTTGGAGGAGTACGGCGGA-3’

*Tu*-62SK **-** F- 5’- CGGGATCCATGGCAGCTCTAGAAAACCA-3’

*Tu*-62SK**-**A -5’- GGAATTCTCATGGGGCGGCCGGAGCGA-3’
