# Supplementary material for: A CsTu‐ TS1 regulatory module promotes fruit tubercule formation in cucumber
Source: Plant Biotechnol J. 2018 Jul 22;17(1):289–301. doi: 10.1111/pbi.12977 (PMC6330641; doi:10.1111/pbi.12977)
Supplement: Supplementary file 3 — Data S1 cDNA and promoter sequences of CsTS1 in different cucumber inbred lines. [file PBI-17-289-s001.docx]

**Supplemental Data. cDNA and promoter sequences of *CsTS1* in different**

**cucumber inbred lines.**

**1.The cDNA sequences of *CsTS1* in different cucumber inbred lines.**

**S1-S22**

ATGGGTGATCGATCACCGCCTCGCCAAGTCCAAGTCCACCCCCAACAACGCTCTTACCTCCAGGACCCCACTTGGAAAATATCCGGTGGCGGCCGCCATGTAGACCACCACCAACATCAAGGCAGCGGCGGCGGCCCCTCCGCTTCGAAAATCATTGCCGTCGTCACCCTCGTCCCCGTTGGTGGCACTCTCCTCGGCCTCTCTGGCCTCACTCTAGCCGCCACGCTCTTCGGTCTTGCTGTGTCGACGCCTGTGTTCCTTCTCTTCAGCCCAGTGATCGTACCAGCTGCGTTAGCAATATGCCTTGCCATCGCTGCATTCTTGACGTCGGGAGTGTTCGGGCTCACAGCGTTGTCGTCACTATCGTGGGTGTATCGGTACATCAGACGGGCGACCGGGACGGTGCCGGAGCAAATGGACATGGCTAAGAGGAAGATGCAGGACATGGCAGGGTATGTGGGACAAAAAACTAAAGAAGTTGGACAAGAAATTCAAAGTAGAACACAAGATCAAGGAAGGAGATCAGGCACAACAGAACAAAGAACTTAA

**L1-L22**

ATGGGTGATCGATCACCGCCTCGCCAAGTCCAAGTCCACCCCCAACAACGCTCTTACCTCCAGGACCCCACTTGGAAAATATCCGGTGGCGGCCGCCATGTAGACCACCACCAACATCAAGGCAGCGGCGGCGGCCCCTCCGCTTCGAAAATCATTGCCGTCGTCACCCTCGTCCCCGTTGGTGGCACTCTCCTCGGCCTCTCTGGCCTCACTCTAGCCGCCACGCTCTTCGGTCTCGCTGTGTCGACGCCTGTGTTCCTTCTCTTCAGCCCAGTGATCGTACCGGCTGCGTTAGCAATATGCCTTGCCATCGCTGCATTCTTGACGTCGGGAGTGTTCGGGCTCACAGCGTTGTCGTCACTATCGTGGGTGTATCGGTACATCAGACGGGCGACCGGGACGGTGCCGGAGCAAATGGACATGGCTAAGAGGAAGATGCAGGACATGGCAGGGTATGTGGGACAAAAAACTAAAGAAGTTGGACAAGAAATTCAAAGTAGAACACAAGATCAAGGAAGGAGATCAGGCACAACAGAACAAAGAACTTAA

**2. The promoter sequences of *CsTS1* in different cucumber inbred lines**

**S1-S22**

GATAGACTTATATCATTGATAGAATTTGACAATTTTTGCTATATTTGTAAATTTTTTTAAATTGTGCTATATACTTAACTAATTTGAATTTAATTGTTAAATTTGCAACTATCCCTTTTTTAATTGTTCTATGAAGACTTCAATTTTAGAGTAATTGTCAATTGCTAACATTTTTAATTAATAATTAAATGTACAATAATTTTTTTTAAAATAGCAAAATCTATAGATGACAAACTCTACTATTAGTGACGTGTACAGATGATATAATATTTACTACGTCATAGTATTTTATAGATCATAGTTTGATAGTTCTACAAATAGTCTACTAAAGTTAATTAATAATATGTATCTATCTTTTAATATAATTATAATGTTTTAACAACTATACTAATTTTTAAAATATCTTATTAAAATTAAAATTTCTAGGGTTTCTTAAAAAACCAATCTTTTGCATAGATAACTGAATATAAAAAAAAGTTAATGCCACCTGGACAAGGTGTGGTTGCTATTAATGCAATGATTTTTTTTTTTGTTATATGATTTTCTATTATATTCATTTTAGCTTAAAAATTGGCCCGTCATCAACGACTCAACCAAGTCCATTGACCAACCAAGTTAACATGAAAATTGTACCAAATAAAAAATTTCATAGTTCATAACTTTTAAACTAGAGATTCTAAATATCATTATGTCAATCAATCATAAGATTCTTTTACTCATCGAATGGAACACTTTGAAAATAGAAATCCAAACATGATAAGTGGCTATATATTTATTGGATGCAAAGCTAGATGTTCTACTAGGGTCCTTGCAAAAGCACACGAGAAAAGCTAACTTAGATCAAACCTAAAACCGATAATGATTACTCAAAGATTCTAGGACACAATTCACTCTAGAATTAGCTTGATCAAAACTAATTAACGAAATTGATTTTAACATCAAATCCAATGCAATATTTGAAAGAAAACACAACATCCACCGGGTAGAATAACATATACATTTTAATATTGATATTATGAGTTTTAGAGATCCTTACAAACGATTTTAAAGGCTCAAATGGTCGTACAAGGAATGGAAACTTAAAATTAATTCAAATATACTAAAATGTCAACTATAGAAAATAATGCCTAGAAACATAAAACACGATATTGCATATTAAATTATAAGCCAAATTGAAAAACAATAAGTGTAAAAAGTTTTCAAAATGTCTTAATTATCTTCTCTAAAATGTGTCAAAGTGGTTGGGACACGATAGACAAGG TTTGACATCACGTTTTTTCATGTTGTGCACAATTGACAACATCTTTCTCACAAAATCTACACATTTTTTTT(T)ATCCTTCGTGTCATCTTCCAAACTATTGGACTTTTTTCGTGTCATCTTTACTTCACTCTCATCTTAACTCATTGAATTAAAGAACATTGTATATTAAATTTAGTTCAAAATTATTTGTTGATGGATGAGAATTTAATAGATTTGAAAGATG--------AAGCGTGTTGGATCTTGTTTATGTTGCTTCTTGTAATTGCTCTTTTGGATACAAGTAATGGATCAATTGATGGAACCACATGAGACTATAACTTTGAATTGAATAAAGAGATGTGTATATTAACATCAAGACAAAATTTGGTCATCTTTTGCACATCCTAACTTGCATTTGATGAAATATTGTCAAAAAATAGTATTATTTTAATATATAT----ATAATTTGTATGATCAAAGAAGGAATGCATAATATTTAGTTTGCCACCTAATCATTTCTCTTCATTATTAAGTTAAAATAAACTC(AC)TTTTAATTATAAGAAACACTCCACTACGAATCTTCTGAATAAATTTAAACAGATACCAAGACAACTATGAATATTGTTTTCTTCCTC---ACAATTTCATGTCTCCAATTTTTTAAAATCCA(CA)GTGTAGAAGTATTCTCAATAATTTAACCAATTAAACGACAATTCTAAATATTTTTATTCAAATATACGAATATGATCTCTTTAAATAGAATTATTATTTTCTTTTTTCATTTTGAACAATGAAACACATAACTTGGAGTGAAACTTGTAGTTCATGCAAACCCTCAACTCTCCAAATCACACATCTACGTGTCACCCTCTTCCCATCTCTACTACTCTTTATATCCTTCCACTTTCCCCTTCTCTTCTCTCTCAACTCCGAACACCGCTCTCCGCCGTACTCCTCCAAC

**L1-L22**

GATAGACTTATATCATTGATAGAATTTGACAATTTTTGCTATATTTGTAAATTTTTTTAAATTGTGCTATATACTTAACTAATTTGAATTTAATTGTTAAATTTGCAACTATCCCTTTTTTAATTGTTCTATGAAGACTTCAATTTTAGAGTAATTGTCAATTGCTAACATTTTTAATTAATAATTAAATGTACAATAATTTTTTTTAAAATAGCAAAATCTATAGATGACAAACTCTACTATTAGTGACGTGTACAGATGATATAATATTTACTACGTCATAGTATTTTATAGATCATAGTTTGATAGTTCTACAAATAGTCTACTAAAGTTAATTAATAATATGTATCTATCTTTTAATATAATTATAATGTTTTAACAACTATACTAATTTTTAAAATATCTTATTAAAATTAAAATTTCTAGGGTTTCTTAAAAAACCAATCTTTTGCATAGATAACTGAATATAAAAAAAAGTTAATGCCACCTGGACAAGGTGTGGTTGCTATTAATGCAATGATTTTTTTTTTTGTTATATGATTTTCTATTATATTCATTTTAGCTTAAAAATTGGCCCGTCATCAACGACTCAACCAAGTCCATTGACCAACCAAGTTAACATGAAAATTGTACCAAATAAAAAATTTCATAGTTCATAACTTTTAAACTAGAGATTCTAAATATCATTATGTCAATCAATCATAAGATTCTTTTACTCATCGAATGGAACACTTTGAAAATAGAAATCCAAACATGATAAGTGGCTATATATTTATTGGATGCAAAGCTAGATGTTCTACTAGGGTCCTTGCAAAAGCACACGAGAAAAGCTAACTTAGATCAAACCTAAAACCGATAATGATTACTCAAAGATTCTAGGACACAATTCACTCTAGAATTAGCTTGATCAAAACTAATTAACGAAATTGATTTTAACATCAAATCCAATGCAATATTTGAAAGAAAACACAACATCCACCGGGTAGAATAACACATACATTTTAATATTGATATTATGAGTTTTAGAGATCCTTACAAACGATTTTAAAGGCTCAAATGGTCGTACAAGAAATGGAAACTTAAAATTAATTCAAATATACTAAAATGTCAACTATAGAAAATAATGCCTATAAACATAAAACACGATATTGCATATTAAATTATAAGCCAAATTGAAAAACAATAAGTGTAAAAAGTTTTCAAAATGTCTTAATTATCTTCTCTAAAATGTGTCAAAGTGGTTGGGACACGATAGACAAGGTTTGACATCACGTTTTTTCATGTTGTGCACAATTGACAACATCTTTCTCACAAAATCTACACATTTTTTTTATCCTTCGTGTCATATTCGAAACTATTGGACTTTTTTCGTGTCATCTTTACTTCATTCTCATCTTAACTCATTGAATTAAAGAACATTGTATATTAAATTTAGTTCAAAATTATTTGTTGATGGATGAGAATTTAATAGATTTGAAAGATGTAGAGTAAAAGCGTGTTGGATCTTGTTTATGTTGCTTCTTGTAATTGCTCTTTTGGATCCAAGTAATGGATCAATTGATGGAACCACATGAGACTATAACTTTGAATTGAATAAAGAGATGTGTATATTAACATCAAGACAAAATTTGGTCATCTTTTGCACATCCTAACTTGCATTTGATGAAATATTGTCAAAAAATAGTATTATTTTAATATATATATATATAATTTGTATGATCAAAGAAGGAATGCATAATATTTAGTTTGCCACCTAATCATTTCTCTTCATTATTAAGTTAAAATAAACTC--TTTTAATTATAAGAGACACTCCACTACGAATCTTCTGAATAAATTTAAACAGATAGCAAGACAACTATGAATATTGTTTTCTTCCTCCTTACAATTTCATGTCTCCAATTTTTTAAAATCCA--GTGTAGAAGTATTCTCAATAATTTAACCAATTAAACGACAATTCTAAATATTTTTATTCAAATATACGAATATGATCTCTTTAAACAGAATTATTATTTTCTTTTTTCATTTTGAACAATGAAACACATAACTTGGAGTGAAACTTGTAGTTCATGCAAACCCTCAACTCTCCAAATCACACATCTACGTGTCACCCTCTTCCCATCTCTACTACTCTTTATATCCTTCCACTTTCCCCTTCACTTCTCTCTCAACTCCGAACACCGCTCTCCGCCGTACTCCTCCAAC
